# Identification of an ergosterol derivative with anti-melanoma effect from the sponge-derived fungus *Pestalotiopsis* sp. XWS03F09

**DOI:** 10.3389/fmicb.2022.1008053

**Published:** 2022-10-12

**Authors:** Tong Xia, Hui Lei, Jianv Wang, Yijing He, Hailan Wang, Lanyang Gao, Tingting Qi, Xia Xiong, Li Liu, Yongxia Zhu

**Affiliations:** ^1^Department of Dermatology, The Affiliated Hospital of Southwest Medical University, Luzhou, China; ^2^School of Pharmacy, Southwest Medical University, Luzhou, China; ^3^Department of Science and Technology, The Affiliated Hospital of Southwest Medical University, Luzhou, China; ^4^School of Public Health, Southwest Medical University, Luzhou, China; ^5^Department of Clinical Pharmacy, Sichuan Cancer Hospital and Institute, Sichuan Cancer Center, School of Medicine, University of Electronic Science and Technology of China, Chengdu, China

**Keywords:** melanoma, ergosterol, antitumor, mitochondrial apoptosis, *OBSCN*

## Abstract

It is difficult to treat malignant melanoma because of its high malignancy. New and effective therapies for treating malignant melanoma are urgently needed. Ergosterols are known for specific biological activities and have received widespread attention in cancer therapy. Here, **LH-1**, a kind of ergosterol from the secondary metabolites of the marine fungus *Pestalotiopsis* sp., was extracted, isolated, purified, and further investigated the biological activities against melanoma. *In vitro* experiments, the anti-proliferation effect on tumor cells was detected by MTT and colony formation assay, and the anti-metastatic effect on tumor cells was investigated by wound healing assay and transwell assay. Subcutaneous xenograft models, histopathology, and immunohistochemistry have been used to verify the anti-tumor, toxic, and side effect *in vivo*. Besides, the anti-tumor mechanism of **LH-1** was studied by mRNA sequencing. *In vitro*, **LH-1** could inhibit the proliferation and migration of melanoma cells A375 and B16-F10 in a dose-dependent manner and promote tumor cell apoptosis through the mitochondrial apoptosis pathway. *In vivo* assays confirmed that **LH-1** could suppress melanoma growth by inducing cell apoptosis and reducing cell proliferation, and it did not have any notable toxic effects on normal tissues. **LH-1** may play an anti-melanoma role by upregulating *OBSCN* gene expression. These findings suggest that **LH-1** may be a potential for the treatment of melanoma.

## Introduction

Malignant melanoma is a kind of cancer with a high incidence, high degree of malignancy, and poor prognosis. It is one of the most difficult to cure malignant tumors in the world. Although it accounts for only 4% of skin cancer cases, it accounts for 75% of skin cancer deaths. The 5-year survival rate is less than 10% (Garbe et al., 2016; Dimitriou et al., 2018; Davis et al., 2019; Suhonen et al., 2021). In China, there are about 20,000 cases of melanoma every year. Compared with Europe and the United States, the incidence rate is slightly lower and has not attracted people’s attention. Most of the newly diagnosed patients have reached the middle and late stages. Subsequently, cancer cells have spread to important organs such as the liver, lung, and brain through blood vessels or lymphatic vessels, of which lung metastasis is the most common site of metastasis (Whiteman et al., 2016; Gershenwald et al., 2017). The treatment for advanced melanoma is limited, in addition to traditional surgical treatment, radiotherapy, and chemotherapy, it also includes targeted therapy and immunotherapy. Although these drugs are still used extensively in clinical treatment, their effect is not satisfactory due to drug resistance and toxic side effects (Ouellet et al., 2016; Grimaldi et al., 2017; Rozeman et al., 2018; Ny et al., 2020; Ziogas et al., 2021). Hence, innovative agents against melanoma are urgently needed. Therefore, the exploration of new drugs for tumor cells that have little effect on normal cells will be beneficial to the treatment of melanoma in the future.

In the last few years, a growing interest in microbial secondary metabolites reflects the importance of microbial secondary metabolites in the discovery and development of new drugs which might be associated with their rich chemical structure (Shen and Thorson, 2012; Wright, 2019; Maglangit et al., 2021). At present, a large number of natural products have been isolated from microbial secondary metabolites and have shown significant biological activities (Yi et al., 2020; Ramirez-Rendon et al., 2022). Among them, marine microorganisms play a major role in the research of natural products, inhabiting environments with extremely high salinity, high pressure, low temperature, and low oxygen for a long time, resulting in a wide variety of secondary metabolites and novel structures. For instance, aspulvinone H isolated from a marine-derived *Aspergillus terreus* showed strong anti-tumor activity in an SW1990-cell-induced xenograft model. Asperphenin A, a lipopeptidyl benzophenone from marine-derived *Aspergillus* sp., has an anti-tumor effect on colon cancer (Cruz et al., 2006; Liu et al., 2016; Bae et al., 2020; Shams Ul Hassan et al., 2021; Yan et al., 2021). As one of the secondary metabolites of fungi, ergosterol analogs have been shown to have anti-tumor, antioxidant, and anti-bacterial properties, and can promote the apoptosis of tumor cells, such as gastric cancer, lung cancer, liver cancer, and breast cancer cells (Shimizu et al., 2016; Tan et al., 2017; Wu et al., 2018; Bu et al., 2019; Zhou et al., 2019). However, the application of ergosterol analogs in anti-melanoma has not been reported in the literature.

*OBSCN* is primarily a gene necessary for the assembly and organization of sarcomere and sarcoplasmic reticulum in the myocardium and skeletal muscle (Young et al., 2001). Obscurins are giant cytoskeletal proteins with structural and regulatory roles which are encoded by the *OBSCN* gene that spans ∼170 kb on human chromosome 1q42 (Young et al., 2001; Ackermann et al., 2014). Although obscurins were originally discovered in striated muscle cells, it is now thought that they are also expressed in non-muscle tissues and play a key role in maintaining cellular homeostasis. A pioneering study that sequenced 13,023 genes in breast and colorectal cancer identified *OBSCN* as one of 189 candidate genes for displaying high-frequency somatic mutations (Sjoblom et al., 2006). Further analysis of the *OBSCN* Gene Mutation Atlas revealed the presence of missense *OBSCN* mutations in melanoma (Balakrishnan et al., 2007). It has been reported that the deletion of the *OBSCN* gene can promote the proliferation and migration of tumor cells in pancreatic cancer and breast cancer (Shriver et al., 2015; Tuntithavornwat et al., 2022).

**FIGURE 1:**
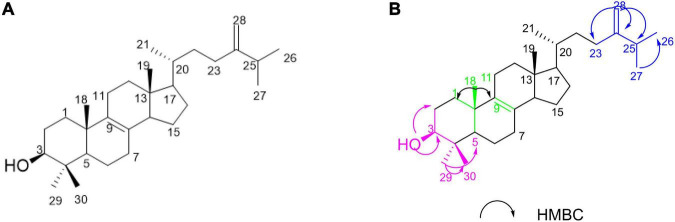
The structure of **LH-1** and the Key HMBC correlations of **LH-1**.

In this study, our investigations on the ethyl acetate (EtOAc) extract of the sponge-derived fungus *Pestalotiopsis* sp. XWS03F09 resulted in the isolation of one compound 4,4-dimethyl-5α-ergosta-8,24(28)-dien-3β-ol (1) (*named* ***LH-1***) (Figure 1A), which was consistent with the compound previously extracted in *Marasmius oreades* (Fattorusso et al., 1992) and *Phycomyces blakesleeanus* (Barrero et al., 1998). However, they only separated, extracted, and identified the compounds, and did not further study the activity and mechanism of the compounds.

**LH-1** was screened for the *in vitro* anticancer activity against various cancer cell lines by the 3-(4,5-dimethyl-2-thiazolyl)-2,5-diphenyltetrazolium bromide (MTT) assay method. We found that melanoma cells were more sensitive to **LH-1** (The IC_50_ value of **LH-1** on B16-F10 melanoma cells was 16.57 μM at 72 h) than other cancer cell lines (IC_50_ values > 60 μM at 72 h). Therefore, we further investigated the biological activities against melanoma *in vitro* and *in vivo*. Our data provided that **LH-1** could inhibit proliferation and migration, induce apoptosis *via* the mitochondria apoptotic pathway, and upregulate *OBSCN* gene expression in melanoma cells. In addition, we also evaluated the anti-tumor activity of **LH-1** in the B16-F10 tumor-bearing xenograft mice model. These results indicate that **LH-1** could be a promising new anti-melanoma drug that is worth further investigation.

## Materials and methods

### Reagents and instruments

AV400MHZ nuclear magnetic resonance spectrometer (Bruker, Germany); BSZ-100 collector (Shanghai Jiapeng Technology Co., Ltd., Shanghai, China); Nmur1300 rotary evaporation instrument (Shanghai Ailang Instrument Co., Ltd., Shanghai, China); ZF-20D dark box ultraviolet spectrometer (Shanghai Baoshan Gucun Electro-Optic Instrument Factory, Shanghai, China); DLSB-5/20 cryogenic coolant circulation instrument (Zhengzhou Science and Technology Industry and Trade Co., Ltd., Zhengzhou, China.); Sephadex-LH-20 (GE Healthcare Bio-sciences AB, Uppsala, Sweden); SW-CJ-2D double purification table (Zhejiang Sujing purification equipment Co., Ltd., Zhejiang, China). Silicone (Anhui Liangchen Silicon Source material Co., Ltd., Anhui, China). 3-(4,5-dimethyl-2-thiazolyl)-2,5-diphenyltetrazolium bromide (MTT, Shanghai Yuan Ye Bio-Technology Co., Ltd., Shanghai, China). Hoechst 33342 solutions (Shanghai Yuanye Technology Co., Ltd.). PI solutions (Beyotime Biotechnology, Shanghai, China). The fluorescence images of cells were obtained by the Nikon Ts2R-FL fluorescence microscope (Tokyo, Japan). The absorbance was determined by SpectraMAX M5 microplate spectrophotometer (Molecular device); Western blot analyses and immunohistochemistry were performed using primary antibodies against Cleaved Caspase 3 antibody, β-actin antibody, and Bcl-2 antibody (Chengdu Zhengneng Biotechnology Co., Ltd., Chengdu, China); Ki-67 antibody, Bax antibody (Cell Signaling Technology, Boston, USA); Cleaved Caspase 9 antibody (Abcam, Shanghai, China).

### Fungal material

The strain was provided by the Yonghong Liu Research Group of the South China Sea Institute of Oceanography, Chinese Academy of Sciences and the preservation number is XWS03F09. The strain was isolated from the sponge *Phakellia fusca* in Xisha Islands and identified as *Pestalotiopsis* sp. by morphological and Internally Transcribed Spacer (ITS) sequence systematic analysis. The strain was preserved in the School of Pharmacy of Southwest Medical University and stored in an MB slant medium at 4°C (Supplementary Table 2).

### Fermentation and isolation

After the strain *Pestalotiopsis* sp. XWS03F09 was activated, and it was inoculated into the plate medium, cultured at 28°C for 5–7 days, inoculated to the liquid medium, and cultured on the 180RPM shaker at 28°C for 2 days, and the seed solution was obtained. A total of 10% concentration was inoculated into the rice solid medium containing 1% crude sea salt and cultured at 28°C for 36 days. The solid strain at the end of fermentation was soaked in acetone, mashed and soaked overnight, filtered with gauze, then extracted with ethyl acetate, concentrated, and the crude substance was obtained. The crude extract of ethyl acetate was eluted by silica gel column chromatography (CH_2_Cl_2_-MeOH) (50:1, 10:1, 5:1, 1:1) to obtain five components of Fr.1∼Fr.5. Three components of Fr.2 were eluted by dextran gel chromatography (Sephadex LH-20 C.C) and eluted with MeOH. The Fr.2.1∼Fr.2.3; Fr.2.2 was eluted by silica gel column chromatography P.E.-EtOAc (Fr.2.1∼Fr.2.3; Fr.2.2 E) (20:1), and the target compound was obtained and named **LH-1**. (220.0 mg).

*4,4-dimethyl-5α-ergosta-8,24(28)-dien-3β-ol (****LH-1****)*, White solid; ${\left[\text{α}\right]}_{\text{D}}^{25}$ 15.9 (c 0.1, MeOH); HRESIMS *m/z* 449.3849 [M+Na]^+^ (Supplementary Figure 1). ^1^H NMR (400 MHz, CDCl_3_) and ^13^C NMR data, see Supplementary Table 3.

### Cell culture

A375, B16-F10, and 293T cells were purchased from the American Type Culture Collection (ATCC, Rockville, MD, USA). They were cultured in DMEM supplemented with 10% heat-inactivated FBS (Gibco, Auckland, New Zealand) and 1% penicillin/streptomycin solution at 37°C in a water-saturated 5% CO_2_ incubator.

### Cell viability assay

Cell viability assay was determined by MTT assay. A375 and B16-F10 cells were seeded overnight in 96-well plates. The cells were treated with different concentrations of **LH-1** (60, 50, 40, 30, 20, 10, 5, 2.5, and 1.25 μM) for 24, 48, and 72 h, respectively. After treatments, 20 μL of 5 mg/mL MTT was added to each well and incubated for 2–4 h. MTT formazan crystals were dissolved in 100 μL DMSO and incubated for 15–20 min. The absorbance at 570 nm was measured by SpectraMAX M5 microplate spectrophotometer (Molecular Devices) and the cell growth survival rate was calculated. Each experiment was performed at least three times.

### Colony formation assay

The A375 and B16-F10 cells were seeded in 6-well plates at 200–500 cells/well and treated with various concentrations of **LH-1** (40, 20, 10, and 2 μM) after 24 h. The cells were incubated for 10 days, and the medicated medium was changed every 3 days. Finally, the cells were fixed with methanol and stained with 0.5% crystal violet for 20 min, and the colonies containing >50 cells were counted. The colony formation numbers were analyzed using Image J software. Each experiment was performed at least three times.

### Wound healing assay

The A375 and B16-F10 cells were seeded in 6-well plates at 3 × 10^5^ cells/well overnight. And the cells were scratched by micro-pipette tips and then washed with PBS twice. And cells were treated with various concentrations of **LH-1** (20, 10, and 2 μM) after 24 h. Then the cells were washed with PBS to remove non-adherent cells and cell debris in the medium. And the migration distance was taken by the microscope and analyzed using Image J software. Each experiment was performed at least three times.

### Transwell migration assay

The migration ability of B16-F10 cells was measured using transwell chambers. Cells in 200 μL serum-free medium with various concentrations of **LH-1** (20 and 2 μM) were added to the upper chamber at a density of 5 × 10^5^/mL, while the lower chamber was filled with 500 μL medium containing 10% FBS. After being treated for 24 h at 37°C, the B16-F10 cells were fixed with methanol for 20 min and stained with crystal violet for 20 min. Finally, cells were viewed by the microscope and analyzed using Image J software. Each experiment was performed at least three times.

### Propidium iodide staining

Propidium iodide (PI) staining was used to observe cell death. The B16-F10 cells (2 × 10^5^ cells/well) were seeded in 6-well plates overnight. After being treated with different concentrations of **LH-1** for 24 h, the B16-F10 cells were washed with PBS and stained with PI solutions. After incubation, the staining effect was observed under the fluorescence microscope.

### Morphological analysis by Hoechst staining

The morphological changes related to apoptosis were observed by Hoechst 33342 staining, and the apoptosis-inducing effect of **LH-1** was detected. After incubating with **LH-1** (40, 20, 10, and 2 μM) for 24 h, the A375 and B16-F10 cells were washed with PBS and stained with Hoechst 33342 solutions (2 μg/mL). Then, the nuclear morphology of cells was observed by fluorescence microscopy.

### Western blot analysis

Collection, lysis, and quantification of B16-F10 cells (BCA Protein Assay Kit; Beyotime, China). Western blot analysis was performed with the indicated antibody. Protein density was analyzed by ImageJ software. The monoclonal antibody against β-actin was used as a control. Data were described as multiple differences from untreated controls.

### Subcutaneous xenograft models

All animal experiments were approved by the Southwest Medical University in China (Permit Number: 201903-38) (Supplementary Table 4), and all protocols were carried out following approved guidelines. Female C57BL/6 mice 4–6 weeks of age were obtained. 5 × 10^5^ cells (100 μL cell suspension) were subcutaneously implanted into the lumbosacral region of C57BL/6 mice to prepare the B16-F10 tumor xenograft model. When the tumors reached an average volume of 100 mm^3^, the mice were randomly divided into three groups (*n* = 6). **LH-1** (10 and 40 mg/kg) or vehicle was administered intraperitoneally once every 2 days for 15 days, and the tumor and body weights were measured every 2 days. The tumor volume was calculated as follows: volume = 0.5 × a × b^2^, among them a (mm) is the length of the tumor, and b (mm) is the width of the tumor. Tumor inhibition rate = (average volume of the control group–average volume of the experimental group)/average volume of the control group ×100%.

### Histopathology and immunohistochemistry

Tumor tissues fixed in formalin were subjected to hematoxylin and eosin (H&E) to detect whether there are any toxic and side effects on normal organs and immunohistochemistry analysis to detect anti-Ki67 and cleaved caspase-3 (Liu et al., 2022).

### mRNA sequencing and bioinformatics analysis

B16-F10 cells treated with **LH-1** for 24 h were sequenced by Shenzhen Huada Genome Co., Ltd., Shenzhen, China. The concentration of **LH-1** was 20 μM. Total RNA was extracted from the cells using Trizol (Invitrogen, Carlsbad, CA, USA) according to manual instruction, qualified and quantified using a NanoDrop and Agilent 2100 bioanalyzer (Thermo Fisher Scientific, Waltham, MA, USA). The sequencing data was filtered with SOAPnuke (v1.5.2) (Li et al., 2008). The heatmap was drawn by heatmap (v1.0.8) according to the gene expression in different samples. Differential expressed genes (DEGs) was identified using DESeq2 package (v1.4.5) (Love et al., 2014) under the criterion of the adjusted *P* < 0.05 and | log_2_FC| > 1. The clusterProfiler Bioconductor software was used to enrich gene ontologies (GO) and the Kyoto encyclopedia of genes and genomes (KEGG) (v4.2.2).

### Quantitative real-time polymerase chain reaction

Cells were lysed with 1 mL of RNA-easy Isolation Reagent (Nanjing Novozan Biotechnology Co., Ltd., Nanjing, China), and cDNA was synthesized with the HiScript^®^ III RT SuperMix for qPCR (+gDNA wiper) (Nanjing Nuowizan Biotechnology Co., Ltd.). QRT-PCR was performed by using Taq Pro Universal SYBR qPCR Master Mix (Nanjing Novozan Biotechnology Co., Ltd.). The Primers used are listed in Supplementary Table 1 of the Supplementary material. Sequence data were submitted to the NCBI Sequence Read Archive under BioProject ID PRJNA866290.

### Statistical analyses

All the results are shown as the mean ± standard deviation (SD), which were determined using GraphPad Prism 8.0 software (GraphPad, Inc., La Jolla, CA, USA). Comparisons between the treatment and negative control groups were conducted by the independent sample *t*-test. Statistically significant *P*-values were labeled as following: *P < 0.05, ^**^P < 0.01, ^***^P < 0.001.

## Results

### Identification of 4,4-dimethyl-5α-ergosta-8,24(28)-dien-3β-ol (LH-1)

**LH-1** was isolated as a white solid. The ^1^H NMR (Supplementary Figure 2) data of **LH-1** displayed seven methyl signals at δ_H_ 0.61 (3H, s, H-18), δ_H_ 1.00 (3H, s, H-19), δ_H_ 0.96 (3H, d, *J* = 6.5 Hz, H-21), δ_H_ 0.99 (3H, d, *J* = 6.5 Hz, H-26), δ_H_ 0.95 (3H, d, *J* = 6.5 Hz, H-27), δ_H_ 0.81 (3H, s, H-29), δ_H_ 1.02 (3H, s, H-30). The ^13^C NMR (Supplementary Figure 3), DEPT (Supplementary Figure 4), and HSQC (Supplementary Figure 5) data showed 30 carbon signals corresponding to seven methyls, 10 methylenes, 6 methines carbons, and 6 oxygenated carbons. A detailed analysis of the NMR data of **LH-1** revealed that it was a known compound, 4,4-dimethyl-5α-ergosta-8,24(28)-dien-3β-ol (**LH-1**), which was previously reported from *P. blakesleeanus*. This was proved by the HMBC (Supplementary Figure 6) correlation from 3-OH (δ_H_ 3.24) to C-29 (δ_C_ 15.4), C-2 (δ_C_ 28.5), C-4 (δ_C_ 39.0), and C-3 (δ_C_ 79.0), from Me-29 (δ_H_ 0.81) to C-30 (δ_C_ 27.9), C-5 (δ_C_ 50.2), C-4 (δ_C_ 39.0), and C-3 (δ_C_ 79.0), from H-25 (δ_H_ 2.21) to C-24 (δ_C_ 156.9), C-28 (δ_C_ 105.9), C-27 (δ_C_ 21.9), and C-26 (δ_C_ 19.9), from H-28 (δ_H_ 4.71) to C-24 (δ_C_ 156.9), C-23 (δ_C_ 31.1), and C-25 (δ_C_ 33.8) (Figure 1B). The planar structure of **LH-1** was determined by HSQC, HMBC, and COSY experiments (Supplementary Figure 7). The NOESY (Supplementary Figures 8, 9) correlations observed between H-3/H-2α; H-1β/H_3_-18; H_3_-18/H-11β; H-H_3_-19 and H-11β; and H-16α/H17α suggested that the hydroxy group on C-3 and the methyl on C-10 had a *cis*-relationship, and the hydroxy at C-3 was in the β-orientation (Supplementary Figure 12). According to the literature and comparison of the NMR data (Barrero et al., 1998), **LH-1** was identified as 4,4-dimethyl-5α-ergosta-8,24(28)-dien-3β-ol.

### LH-1 inhibited the proliferation of melanoma cells

**FIGURE 2:**
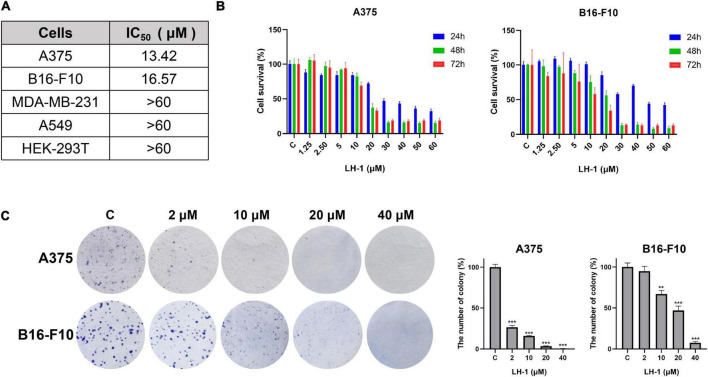
**LH-1** inhibited melanoma cell viability. **(A)** IC_50_ of **LH-1** in melanoma cells A375, B16-F10, human breast cancer cells MDA-MB-231, human lung adenocarcinoma cells A549, and human renal epithelial cells HEK-293T for 72 h. **(B)** Melanoma cell lines A375 and B16-F10 were treated with different concentrations of **LH-1** for 24, 48, or 72 h, respectively. And the viability was measured by the MTT assay. **(C)** The effects of **LH-1** on colony formation in two melanoma cell lines for 10 days and the statistical data were shown on the right. All experiments were repeated at least three times. **P < 0.01, ***P < 0.001 vs. control(C).

The MTT method was used to detect the anti-proliferative activity of **LH-1** on different tumor cells. It was found that **LH-1** had a selective inhibitory effect on melanoma cells and had no significant toxicity to human breast cancer cell MDA-MB-231 (IC_50_ > 60 μM at 72 h), human carcinoma cell A549 (IC_50_ > 60 μM at 72 h) and human renal epithelial cell HEK-293T (IC_50_ > 50 μM at 72 h) (Figure 2A and Supplementary Figure 10). The A375 and B16-F10 cells were treated with **LH-1** at different concentrations for 24, 48, and 72 h. As shown in Figure 2B, the inhibitory effect of **LH-1** on the proliferation of melanoma cells in a concentration and time-dependent manner. The IC_50_ values of **LH-1** on A375 and B16-F10 cells melanoma cells were 13.42 and 16.57 μM at 72 h, respectively. Furthermore, we did colony-forming assays to further investigate the effect of **LH-1** on the A375 and B16-F10 cells. After treatment with different concentrations of **LH-1**, the colony formation numbers of A375 and B16-F10 in the 10 μM group were 15.92 and 66.9% of those in the control group, and 0.55 and 7.46% in the 40 μM group, respectively (Figure 2C).

### LH-1 inhibited the migration of melanoma cells

**FIGURE 3:**
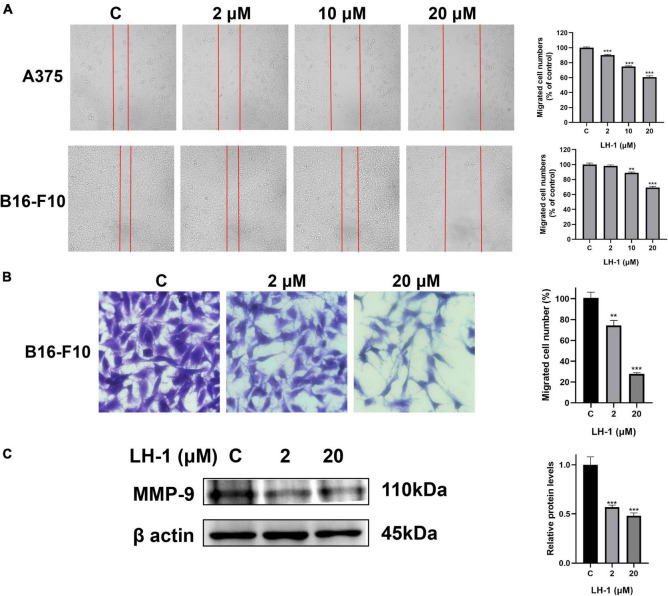
**LH-1** inhibited melanoma cells A375 and B16-F10 migration. **(A)** A single scratch was created in the confluent monolayer of A375 and B16-F10 cells. The scratch was photographed at 24 h after **LH-1** treatment (left) and migrated cell numbers (right) were analyzed by Image J software. **(B)** Cells were allowed to migrate through the transwell chamber in the presence of an indicated concentration of **LH-1**. Representative photographs of migrated cells (left) and quantification of these cells (right) were shown. **(C)** Cells were treated with the indicated concentration of **LH-1** for 24 h, expression levels of MMP-9 were determined by the Western blot analysis (left), and relative expression levels were analyzed by Image J software (right). All experiments were repeated at least three times. **P < 0.01, ***P < 0.001 vs. control.

The colony formation results agree with the MTT data and indicated that **LH-1** selectively inhibited the growth of melanoma cells. In addition, the cell wound-healing, transwell assay, and western blot assay were performed to evaluate the anti-metastatic effect of **LH-1**. As shown in Figure 3A, the inhibitory effect increased with the increase in concentration. The migration capacity of A375 and B16-F10 in the 10 μM group was 74.79 and 88.76% of that in the control group, and 60.52 and 69.04% in the 20 μM group, respectively. The transwell assay results showed that the number of cells passing through the transwell chamber in the **LH-1** group was lower than that in the control group. **LH-1** significantly inhibited transwell migration in A375 and B16-F10 melanoma cells in a dose-dependent manner (Figure 3B). And the MMP-9 showed an apparent decrease after treatment of **LH-1** (Figure 3C). To sum up, this finding suggested that **LH-1** could inhibit the proliferation and migration of melanoma cells.

### LH-1 induced apoptosis by the mitochondrial apoptotic pathway in melanoma cells

**FIGURE 4:**
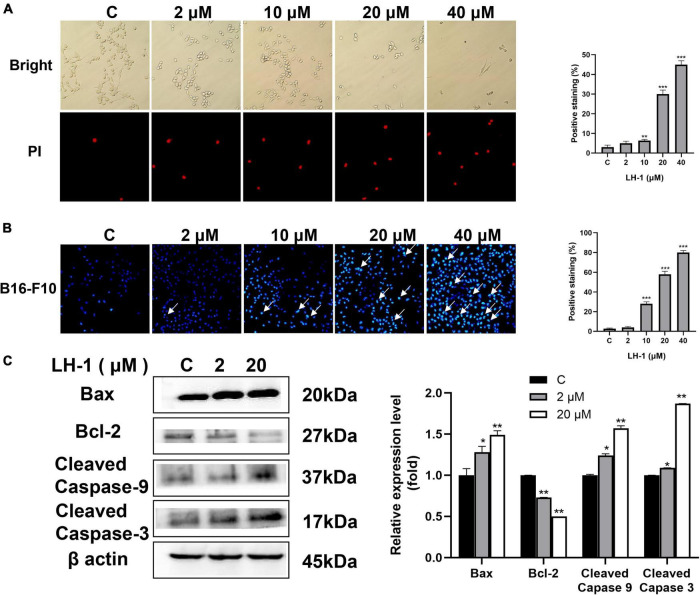
**LH-1** induced cell apoptosis *via* mitochondria-mediated apoptosis. **(A)** B16-F10 cells were treated with the indicated concentration of **LH-1** for 24 h, and the morphology of cells and the cell with positive propidium iodide (PI) staining were under a fluorescence microscope. **(B)** Hoechst staining was used to stain apoptotic cells after treatment with **LH-1**. **(C)** Cells were treated with the indicated concentration of **LH-1** for 24 h, and the expression levels of Bax, Bcl-2, Cleaved Caspase-3, and Cleaved Caspase-9 were determined by the Western blot analysis (left), and relative expression levels were analyzed by Image J software (right). All experiments were repeated at least three times. *P < 0.05, **P < 0.01, ***P < 0.001 vs. control.

Avoiding apoptosis is one of the signs of cancer (Hanahan and Weinberg, 2011). Mitochondrial apoptosis is one of the main pathways of cancer cell death. Apoptosis is characterized by cell shrinkage, chromatin condensation, and nuclear and cell fragmentation. These characteristics lead to the formation of apoptotic bodies (Cotter, 2009). As shown in Figure 4A, with the increase in **LH-1** concentration, the number of remaining cells gradually decreased and the morphology changed significantly. At the same time, the number of PI-positive cells gradually increased. To evaluate whether **LH-1** can induce apoptosis, Hoechst 33342 staining was performed to visualize the formation of apoptotic bodies. As shown in Figure 4B and Supplementary Figure 11, apoptotic bodies and chromatin agglutination are significantly increased compared with control. Besides, these changes were concentration-dependent. Anti-apoptotic members of the Bcl-2 family such as pro-apoptotic proteins Bax and anti-apoptotic proteins Bcl-2 are representative markers of apoptosis (Cory et al., 2003). To further confirm the apoptosis-promoting effect of **LH-1**, the expression of apoptosis-related proteins was detected by western blot assay. We examined Bax, Bcl-2, Cleaved Caspase-3, and Cleaved Caspase-9 expression levels in B16-F10 cells after treatment with **LH-1** for 24 h. As shown in Figure 4C, the expression level of Bcl-2 was significantly decreased, whereas Bax, Cleaved Caspase-3, and Cleaved Caspase-9 were increased in a dose-dependent manner. These results suggested that the inhibitory effect of **LH-1** on melanoma cells is achieved by inducing the mitochondrial apoptotic pathway.

### LH-1 inhibited melanoma tumor growth *in vivo*

**FIGURE 5:**
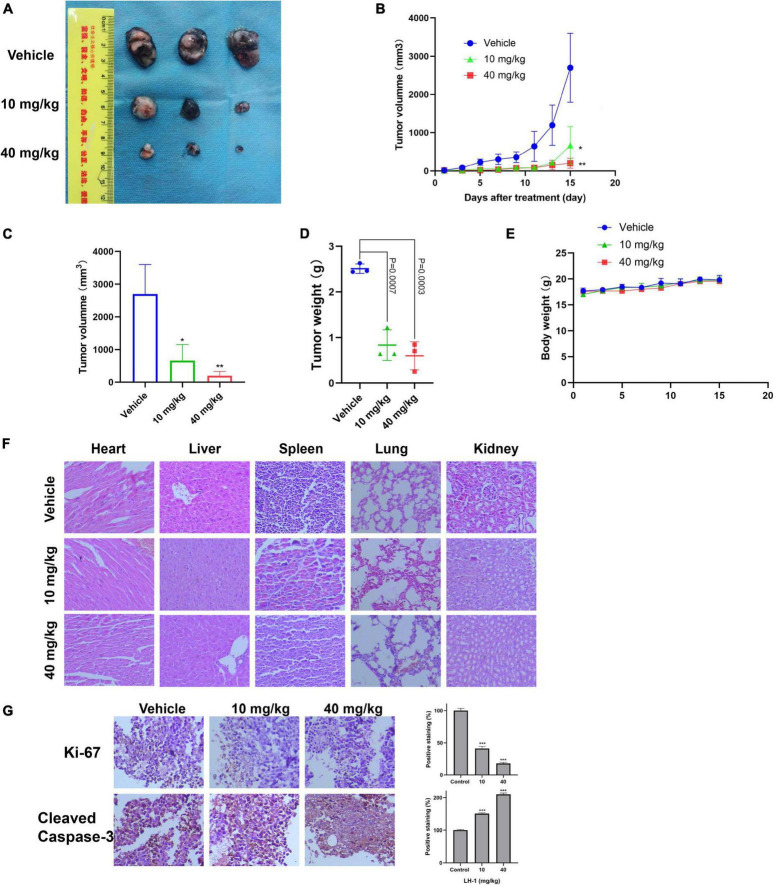
**LH-1** inhibited tumor growth *in vivo*. In the B16-F10 xenograft model, the mice were treated with **LH-1** (10 and 40 mg/kg) or vehicle. **(A)** Tumors from mice treated with **LH-1** or vehicle on the final day. **(B)** Tumor volumes were measured every 2 days and were represented as the mean ± SD (*n* = 6, *P < 0.05, **P < 0.01 versus vehicle group). **(C)** Quantitative analysis of tumor volume changes on the final day (Day 15). **(D)** Represented the weight of tumors from mice in different groups when the mice were sacrificed at the endpoint. *P*-values for comparing two groups were determined using a two-tailed Student’s *t*-test. **(E)** Body weights were measured every 2 days and were represented as the mean ± SD (*n* = 3, The difference was not statistically significant). **(F)** Histological observation of **LH-1** treated mice. Microscopic pathology of the heart, liver, spleen, lung, and kidney shows no evidence of adverse systemic toxicity following **LH-1** treatment in the mice. Slides were observed under a microscope (×200). **(G)** Tumor cell proliferation was evaluated through immunohistochemical analysis staining with Ki-67 and cleaved caspase-3, and the statistical data of positive staining were shown on the right. *P < 0.05, **P < 0.01, ***P < 0.001 vs. vehicle.

To test the anti-tumor effect of **LH-1** *in vivo*, the xenograft model of B16-F10 cells in mice was established and the mice were injected intraperitoneally with 10 and 40 mg/kg every other day for 15 days. As shown in Figures 5A–D, compared with the vehicle group, **LH-1** treatment could inhibit tumor growth and tumor weight, and the inhibitory effect of the 40 mg/kg dose group was stronger than that of the 10 mg/kg dose group. The tumor inhibition rate of the low-dose group is 75.44%, and the high-dose group is 92.56%. Notably, during treatment, there was no significant change in the body weight of mice during the treatment (Figure 5E). The above data indicated that **LH-1** inhibited melanoma tumor growth *in vivo* and has no significant effect on the body weight of mice. To validate whether **LH-1** is toxic to normal organs of mice, the main organs were stained with hematoxylin and eosin (H&E) staining and observed under a light microscope. No histopathological abnormality was found in the treatment group (Figure 5F), these results indicated that **LH-1** had no significant toxic or side effects on the main organs of mice. To further understand the anti-tumor mechanism of **LH-1** *in vivo*, immunohistochemistry analysis was performed. As is shown in Figure 5G, after **LH-1** treatment, cell proliferation (Ki-67 positive) significantly decreased and apoptosis significantly increased (Cleaved Caspase-3 positive). Consequently, the **LH-1** inhibited tumor growth in the B16-F10 xenograft tumor model by inhibiting cell proliferation and inducing cell apoptosis.

### LH-1 induces *OBSCN* expression

**FIGURE 6:**
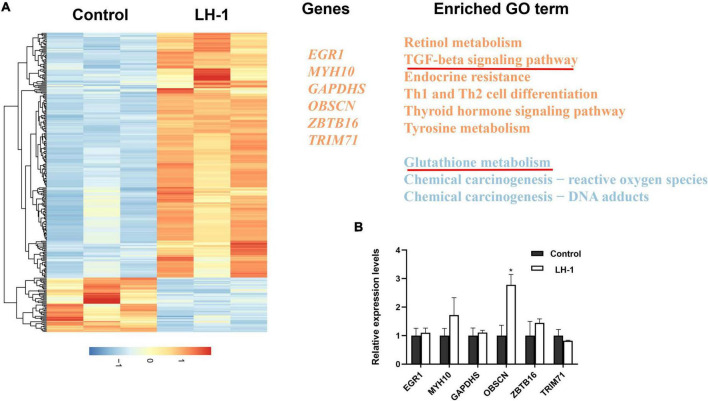
Gene expression changes induced by **LH-1** in melanoma cells. **(A)** Differential expressed genes (DEGs) expression profile and related functional term enrichments. Each row represents a single DEG and each column represents a sample. Heatmap values were normalized from –2 (blue; low expression counts) to +2 (red; high expression counts) (the left heatmap panel). Representative genes (middle panel) and enriched pathways (right panel) are then displayed. The upregulated genes and pathways are marked by orange, and the downregulated pathways are marked by blue. **(B)** The qRT-PCR validation of representative gene expression in B16-F10 cells. qRT-PCR was repeated at least three times. *P < 0.05 vs. control.

To further study the anti-melanoma mechanism of **LH-1**, mRNA sequencing was performed in B16-F10 cells treated with **LH-1**. As shown in Figure 6A, the total number of 6,130 genes was markedly altered in B16-F10 cells, a heatmap was generated to show **LH-1**-induced changes in gene expression in B16-F10 cells. The GO enrichment analysis indicated that the significant changes mainly focused on the “TGF-β signaling pathway” and “Glutathione transferase activity.” As we all know, the transforming growth factor (TGF)-β signaling events are to control diverse processes and numerous responses, such as cell proliferation, differentiation, apoptosis, and migration (Xie et al., 2018). Among them, promoting apoptosis and mesenchymal transformation are the two most important functions of TGF-β (Song and Shi, 2018). Although our experimental results showed that the TGF-β signaling pathway was activated after **LH-1** treatment, the occurrence and development of the tumor is a complex process, not the result of the action of a single factor. In the future, the specific mechanisms of action can be studied by detecting key proteins in this pathway. In addition, γ-Glutamylcyclotransferase is one of the main enzymes in glutathione metabolism. It is upregulated in many cancers (breast, ovarian, cervical, lung, etc.) and promotes the progression of cancer. Its deletion leads to the inhibition of cancer cell proliferation, invasion, and migration (Kageyama et al., 2018). The experimental results suggested that **LH-1** may inhibit melanoma growth by downregulating glutathione metabolism. And then several genes related to carcinogenesis were selected from the top 20 mRNA-seq upregulated genes and were further verified by qRT-PCR. As shown in Figure 6B, the *OBSCN* gene was upregulated significantly. Our study suggested that **LH-1** may play an anti-melanoma role by upregulating *OBSCN* gene expression, activating the TGF-β signaling pathway, and inhibiting glutathione transferase activity.

## Discussion

Human malignant melanoma is a highly invasive human cancer, and its incidence has increased faster than any other cancer in the past few decades (Sung et al., 2021). For advanced melanoma, targeted therapies (e.g., dabrafenib, ipilimumab) are now available in other countries but are costly (Gordon et al., 2016). Therefore, new anti-melanoma targeted therapies are urgently needed. In recent years, due to the wide sources of natural products, more and more studies have been conducted on their anti-tumor effects (Fontana et al., 2019). Especially, microbial secondary metabolites have become a rich source of new drugs, which have made great contributions to the discovery of new drugs (Lei et al., 2021; Ramirez-Rendon et al., 2022).

**FIGURE 7:**
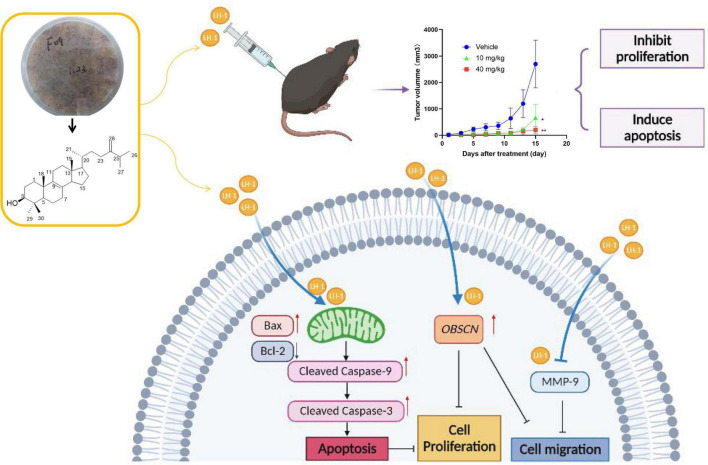
Diagram of the mechanism of LH-1 inhibiting melanoma.

In this study, we extracted, isolated, and purified the target compound **LH-1** from the secondary metabolites of the marine fungus *Pestalotiopsis* sp. and its structure was identified as 4,4-dimethyl-5α-ergosta-8,24(28)-dien-3β-ol. Afterward, its biological activities against melanoma were explored for the first time. The inhibitory effect of **LH-1** on melanoma and its molecular mechanism was investigated *in vitro* and *in vivo* (Figure 7). First of all, **LH-1** inhibited the proliferation of two melanoma cell lines in a time-and concentration-dependent manner and had no significant inhibitory effect on normal cells. A sterol derived ergosterol peroxide (EP), extracted from medicinal mushrooms, has been reported to exert antitumor activity in several tumor types. The IC_50_ to ovarian cancer cells and lung cancer cells is about 50 and 23 μM, respectively (Tan et al., 2017; Wu et al., 2018). However, the IC_50_ value of **LH-1** on B16-F10 melanoma cells is 16.57 μM, which is better than that of EP. Furthermore, to explore the anti-proliferation mechanism of **LH-1**, Hoechst 33342 staining was performed, and we found that **LH-1** could induce apoptosis of melanoma cells in a dose-dependent manner. Apoptosis is an important way to destroy cancer cells, and the mitochondrial apoptosis pathway is the main signal pathway of apoptosis (Cotter, 2009). Bax, a member of the pro-apoptotic family, is a member of the BCL-2 family, which plays a central role in the permeability of the mitochondrial outer membrane and then induces apoptosis (Gibson and Davids, 2015). After **LH-1** treatment, the pro-apoptotic protein Bax, Cleaved Caspase-3, and Cleaved Caspase-9 were increased, while the anti-apoptotic proteins Bcl-2 were decreased. Summarizing these data, **LH-1** induced the apoptosis of melanoma cells through the mitochondrial apoptotic pathway. *In vivo*, the C57BL/6 mice tumor model of subcutaneous transplants showed that **LH-1** inhibited tumor growth and had no significant effect on the body weight of mice. We also found the activation of apoptosis (Cleaved Caspase-3 positive cells) and the decrease of cell proliferation (Ki-67 positive cells) by immunohistochemistry with mouse tumor tissue.

The main cause of melanoma death is the occurrence of metastases (Xia et al., 2021). Upregulation of MMP-9 is often associated with increased cancer cell migration, invasion, and metastasis (Zhu et al., 2019). Therefore, the inhibitory effect of **LH-1** on melanoma metastasis was evaluated by cell migration assay. Wound healing assay and transwell assay showed that **LH-1** could inhibit the migration of melanoma cells. In addition, **LH-1** downregulated the expression levels of MMP-9 in B16-F10. The above results have shown that **LH-1** inhibited the migration of melanoma *in vitro*.

Recently, *OBSCN* mutations have been found in many types of cancer, suggesting that the gene may play a key role in different cancers. It has been reported that giant obscurin regulates migration and metastasis in pancreatic cancer (Tuntithavornwat et al., 2022), and the loss of giant obscurins from breast epithelium promotes epithelial-to-mesenchymal transition, tumorigenicity, and metastasis (Shriver et al., 2015). These results suggest that *OBSCN* may be a gene related to inhibiting the occurrence and development of cancer. Some studies have found that *OBSCN* has mutations in melanoma, suggesting that *OBSCN* may be related to the mechanism of melanoma progression (Balakrishnan et al., 2007). We found that the *OBSCN* was significantly increased after **LH-1** treatment in melanoma cells, and it was speculated that **LH-1** inhibited the proliferation and migration of melanoma by targeting *OBSCN*. However, how **LH-1** affects the upregulation of *OBSCN* requires further analysis. (Tan et al., 2017) reported that EP showed antitumor effects toward ovarian cancer cells through both β-catenin and STAT3 signaling pathways, and we speculate whether **LH-1**, which is similar to EP’s structure, also acts through this pathway. We will study in this direction in follow-up experiments.

Due to the easy access and non-toxicity side effects, the use of marine natural products particularly marine fungi-derived biomolecules for inflammation, and cancer therapy has received much attention. This study paves the way for us to study anti-melanoma drugs of marine fungal secondary metabolites.

## Conclusion

This study has extracted, isolated, purified the target compound **LH-1**, and evaluated the anti-melanoma activity of **LH-1** *in vivo* and *in vitro*. Our study verified that **LH-1** induced apoptosis through the mitochondrial apoptosis pathway and inhibited the migration *via* downregulated expression levels of MMP-9 in melanoma cells. In addition, **LH-1** showed anti-tumor activity in the xenograft model *in vivo* and had no significant toxicity. Altogether, **LH-1** is a promising candidate for treating melanoma growth and metastasis.

## Data availability statement

The datasets presented in this study can be found in online repositories. The names of the repository/repositories and accession number(s) can be found below: PRJNA866290 (BioProject ID).

## Ethics statement

The animal study was reviewed and approved by Southwest Medical University in China (Permit Number: 201903-38).

## Author contributions

LL, HL, and TX designed the study. TX, HL, YZ, and LL wrote the manuscript. TX, HL, JW, and HW performed the experiments. TX, LG, and XX analyzed the data. TX, HL, and TQ revised the manuscript. All authors have read and agreed to the published version of the manuscript.

## References

[B1] Ackermann M. A. Shriver M. Perry N. A. Hu L. Y. Kontrogianni-Konstantopoulos A. (2014). Obscurins: Goliaths and Davids take over non-muscle tissues. *PLoS One* 9:e88162. 10.1371/journal.pone.0088162 24516603 PMC3916441

[B2] Bae S. Y. Liao L. Park S. H. Kim W. K. Shin J. Lee S. K. (2020). Antitumor activity of asperphenin A, a lipopeptidyl benzophenone from marine-derived *Aspergillus* sp. Fungus, by inhibiting tubulin polymerization in colon cancer cells. *Mar. Drugs* 18:110. 10.3390/md18020110 32069904 PMC7073961

[B3] Balakrishnan A. Bleeker F. E. Lamba S. Rodolfo M. Daniotti M. Scarpa A. (2007). Novel somatic and germline mutations in cancer candidate genes in glioblastoma, melanoma, and pancreatic carcinoma. *Cancer Res.* 67 3545–3550. 10.1158/0008-5472.CAN-07-0065 17440062

[B4] Barrero A. F. Oltra J. E. Poyatos J. A. Jiménez D. Oliver E. (1998). Phycomysterols and other sterols from the fungus *Phycomyces blakesleeanus*. *J. Nat. Prod.* 61 1491–1496. 10.1021/np980199h 9868149

[B5] Bu M. Li H. Wang H. Wang J. Lin Y. Ma Y. (2019). Synthesis of ergosterol peroxide conjugates as mitochondria targeting probes for enhanced anticancer activity. *Molecules* 24:3307. 10.3390/molecules24183307 31514398 PMC6766909

[B6] Cory S. Huang D. C. Adams J. M. (2003). The Bcl-2 family: Roles in cell survival and oncogenesis. *Oncogene* 22 8590–8607. 10.1038/sj.onc.1207102 14634621

[B7] Cotter T. G. (2009). Apoptosis and cancer: The genesis of a research field. *Nat. Rev. Cancer* 9 501–507. 10.1038/nrc2663 19550425

[B8] Cruz L. J. Insua M. M. Baz J. P. Trujillo M. Rodriguez-Mias R. A. Oliveira E. (2006). IB-01212, a new cytotoxic cyclodepsipeptide isolated from the marine fungus *Clonostachys* sp. ESNA-A009. *J. Org. Chem.* 71 3335–3338. 10.1021/jo051600p 16626111

[B9] Davis L. E. Shalin S. C. Tackett A. J. (2019). Current state of melanoma diagnosis and treatment. *Cancer Biol. Ther.* 20 1366–1379. 10.1080/15384047.2019.1640032 31366280 PMC6804807

[B10] Dimitriou F. Krattinger R. Ramelyte E. Barysch M. J. Micaletto S. Dummer R. (2018). The world of melanoma: Epidemiologic, genetic, and anatomic differences of melanoma across the globe. *Curr. Oncol. Rep.* 20:87. 10.1007/s11912-018-0732-8 30250984

[B11] Fattorusso E. Giovannitti B. Lanzotti V. Magno S. Violante U. (1992). 4, 4-Dimethyl-5α-ergosta-8,24(28)dien-3β-ol from the fungus *Marasmius oreades*. *Steroids* 57 119–121. 10.1016/0039-128x(92)90069-l 1621266

[B12] Fontana F. Raimondi M. Di Domizio A. Moretti R. M. Montagnani Marelli M. Limonta P. (2019). Unraveling the molecular mechanisms and the potential chemopreventive/therapeutic properties of natural compounds in melanoma. *Semin. Cancer Biol.* 59 266–282. 10.1016/j.semcancer.2019.06.011 31233829

[B13] Garbe C. Peris K. Hauschild A. Saiag P. Middleton M. Bastholt L. (2016). Diagnosis and treatment of melanoma. European consensus-based interdisciplinary guideline - Update 2016. *Eur. J. Cancer* 63 201–217. 10.1016/j.ejca.2016.05.005 27367293

[B14] Gershenwald J. E. Scolyer R. A. Hess K. R. Sondak V. K. Long G. V. Ross M. I. (2017). Melanoma staging: Evidence-based changes in the American Joint Committee on Cancer eighth edition cancer staging manual. *CA Cancer J. Clin.* 67 472–492. 10.3322/caac.21409 29028110 PMC5978683

[B15] Gibson C. J. Davids M. S. (2015). BCL-2 antagonism to target the intrinsic mitochondrial pathway of apoptosis. *Clin. Cancer Res.* 21 5021–5029. 10.1158/1078-0432.CCR-15-0364 26567361 PMC4646729

[B16] Gordon L. G. Elliott T. M. Wright C. Y. Deghaye N. Visser W. (2016). Modelling the healthcare costs of skin cancer in South Africa. *BMC Health Serv. Res.* 16:113. 10.1186/s12913-016-1364-z 27039098 PMC4818961

[B17] Grimaldi A. M. Simeone E. Festino L. Vanella V. Strudel M. Ascierto P. A. (2017). MEK inhibitors in the treatment of metastatic melanoma and solid tumors. *Am. J. Clin. Dermatol.* 18 745–754. 10.1007/s40257-017-0292-y 28537004

[B18] Hanahan D. Weinberg R. A. (2011). Hallmarks of cancer: The next generation. *Cell* 144 646–674. 10.1016/j.cell.2011.02.013 21376230

[B19] Kageyama S. Ii H. Taniguchi K. Kubota S. Yoshida T. Isono T. (2018). Mechanisms of tumor growth inhibition by depletion of gamma-glutamylcyclotransferase (GGCT): A novel molecular target for anticancer therapy. *Int. J. Mol. Sci.* 19:2054. 10.3390/ijms19072054 30011933 PMC6073726

[B20] Lei H. Bi X. Lin X. She J. Luo X. Niu H. (2021). Heterocornols from the sponge-derived fungus pestalotiopsis heterocornis with anti-inflammatory activity. *Mar. Drugs* 19:585. 10.3390/md19110585 34822456 PMC8620458

[B21] Li R. Li Y. Kristiansen K. Wang J. (2008). SOAP: Short oligonucleotide alignment program. *Bioinformatics* 24 713–714. 10.1093/bioinformatics/btn025 18227114

[B22] Liu H. Li X. M. Liu Y. Zhang P. Wang J. N. Wang B. G. (2016). Chermesins A-D: Meroterpenoids with a drimane-type spirosesquiterpene skeleton from the marine algal-derived endophytic fungus *Penicillium chermesinum* EN-480. *J. Nat. Prod.* 79 806–811. 10.1021/acs.jnatprod.5b00893 26990653

[B23] Liu Z. Wang H. Sun C. He Y. Xia T. Wang J. (2022). ZWZ-3, a fluorescent probe targeting mitochondria for melanoma imaging and therapy. *Front. Pharmacol.* 13:829684. 10.3389/fphar.2022.829684 35281928 PMC8905922

[B24] Love M. I. Huber W. Anders S. (2014). Moderated estimation of fold change and dispersion for RNA-seq data with DESeq2. *Genome Biol.* 15:550. 10.1186/s13059-014-0550-8 25516281 PMC4302049

[B25] Maglangit F. Yu Y. Deng H. (2021). Bacterial pathogens: Threat or treat (a review on bioactive natural products from bacterial pathogens). *Nat. Prod. Rep.* 38 782–821. 10.1039/d0np00061b 33119013

[B26] Ny L. Hernberg M. Nyakas M. Koivunen J. Oddershede L. Yoon M. (2020). BRAF mutational status as a prognostic marker for survival in malignant melanoma: A systematic review and meta-analysis. *Acta Oncol.* 59 833–844. 10.1080/0284186X.2020.1747636 32285732

[B27] Ouellet D. Kassir N. Chiu J. Mouksassi M. S. Leonowens C. Cox D. (2016). Population pharmacokinetics and exposure-response of trametinib, a MEK inhibitor, in patients with BRAF V600 mutation-positive melanoma. *Cancer Chemother. Pharmacol.* 77 807–817. 10.1007/s00280-016-2993-y 26940938

[B28] Ramirez-Rendon D. Passari A. K. Ruiz-Villafan B. Rodriguez-Sanoja R. Sanchez S. Demain A. L. (2022). Impact of novel microbial secondary metabolites on the pharma industry. *Appl. Microbiol. Biotechnol.* 106 1855–1878. 10.1007/s00253-022-11821-5 35188588 PMC8860141

[B29] Rozeman E. A. Dekker T. J. A. Haanen J. Blank C. U. (2018). Advanced melanoma: Current treatment options, biomarkers, and future perspectives. *Am. J. Clin. Dermatol.* 19 303–317. 10.1007/s40257-017-0325-6 29164492

[B30] Shams Ul Hassan S. Ishaq M. Zhang W. D. Jin H. Z. (2021). An overview of the mechanisms of marine fungi-derived anti-inflammatory and anti-tumor agents and their novel role in drug targeting. *Curr. Pharm. Des.* 27 2605–2614. 10.2174/1381612826666200728142244 32723250

[B31] Shen B. Thorson J. S. (2012). Expanding nature’s chemical repertoire through metabolic engineering and biocatalysis. *Curr. Opin. Chem. Biol.* 16 99–100. 10.1016/j.cbpa.2012.03.006 22464247

[B32] Shimizu T. Kawai J. Ouchi K. Kikuchi H. Osima Y. Hidemi R. (2016). Agarol, an ergosterol derivative from *Agaricus blazei*, induces caspase-independent apoptosis in human cancer cells. *Int. J. Oncol.* 48 1670–1678. 10.3892/ijo.2016.3391 26893131

[B33] Shriver M. Stroka K. M. Vitolo M. I. Martin S. Huso D. L. Konstantopoulos K. (2015). Loss of giant obscurins from breast epithelium promotes epithelial-to-mesenchymal transition, tumorigenicity and metastasis. *Oncogene* 34 4248–4259. 10.1038/onc.2014.358 25381817 PMC4426246

[B34] Sjoblom T. Jones S. Wood L. D. Parsons D. W. Lin J. Barber T. D. (2006). The consensus coding sequences of human breast and colorectal cancers. *Science* 314 268–274. 10.1126/science.1133427 16959974

[B35] Song J. Shi W. (2018). The concomitant apoptosis and EMT underlie the fundamental functions of TGF-beta. *Acta Biochim. Biophys. Sin.* 50 91–97. 10.1093/abbs/gmx117 29069287

[B36] Suhonen V. Rummukainen J. Siiskonen H. Mannermaa A. Harvima I. T. (2021). High regional mortality due to malignant melanoma in Eastern Finland may be explained by the increase in aggressive melanoma types. *BMC Cancer* 21:1155. 10.1186/s12885-021-08879-1 34711205 PMC8555296

[B37] Sung H. Ferlay J. Siegel R. L. Laversanne M. Soerjomataram I. Jemal A. (2021). Global cancer statistics 2020: GLOBOCAN estimates of incidence and mortality worldwide for 36 cancers in 185 countries. *CA Cancer J. Clin.* 71 209–249. 10.3322/caac.21660 33538338

[B38] Tan W. Pan M. Liu H. Tian H. Ye Q. Liu H. (2017). Ergosterol peroxide inhibits ovarian cancer cell growth through multiple pathways. *Onco Targets Ther.* 10 3467–3474. 10.2147/OTT.S139009 28761355 PMC5518915

[B39] Tuntithavornwat S. Shea D. J. Wong B. S. Guardia T. Lee S. J. Yankaskas C. L. (2022). Giant obscurin regulates migration and metastasis via RhoA-dependent cytoskeletal remodeling in pancreatic cancer. *Cancer Lett.* 526 155–167. 10.1016/j.canlet.2021.11.016 34826548 PMC9427004

[B40] Whiteman D. C. Green A. C. Olsen C. M. (2016). The growing burden of invasive melanoma: Projections of incidence rates and numbers of new cases in six susceptible populations through 2031. *J. Invest. Dermatol.* 136 1161–1171. 10.1016/j.jid.2016.01.035 26902923

[B41] Wright G. D. (2019). Unlocking the potential of natural products in drug discovery. *Microb. Biotechnol.* 12 55–57. 10.1111/1751-7915.13351 30565871 PMC6302737

[B42] Wu H. Y. Yang F. L. Li L. H. Rao Y. K. Ju T. C. Wong W. T. (2018). Ergosterol peroxide from marine fungus *Phoma* sp. induces ROS-dependent apoptosis and autophagy in human lung adenocarcinoma cells. *Sci. Rep.* 8:17956. 10.1038/s41598-018-36411-2 30560887 PMC6298985

[B43] Xia Y. Xu F. Xiong M. Yang H. Lin W. Xie Y. (2021). Repurposing of antipsychotic trifluoperazine for treating brain metastasis, lung metastasis and bone metastasis of melanoma by disrupting autophagy flux. *Pharmacol. Res.* 163:105295. 10.1016/j.phrs.2020.105295 33176207

[B44] Xie F. Ling L. van Dam H. Zhou F. Zhang L. (2018). TGF-beta signaling in cancer metastasis. *Acta Biochim. Biophys. Sin.* 50 121–132. 10.1093/abbs/gmx123 29190313

[B45] Yan S. Qi C. Song W. Xu Q. Gu L. Sun W. (2021). Discovery of GOT1 inhibitors from a marine-derived *Aspergillus terreus* that act against pancreatic ductal adenocarcinoma. *Mar. Drugs* 19:588. 10.3390/md19110588 34822459 PMC8618880

[B46] Yi M. Lin S. Zhang B. Jin H. Ding L. (2020). Antiviral potential of natural products from marine microbes. *Eur. J. Med. Chem.* 207:112790. 10.1016/j.ejmech.2020.112790 32937282 PMC7457942

[B47] Young P. Ehler E. Gautel M. (2001). Obscurin, a giant sarcomeric Rho guanine nucleotide exchange factor protein involved in sarcomere assembly. *J. Cell Biol.* 154 123–136. 10.1083/jcb.200102110 11448995 PMC2196875

[B48] Zhou B. Liang X. Feng Q. Li J. Pan X. Xie P. (2019). Ergosterol peroxide suppresses influenza A virus-induced pro-inflammatory response and apoptosis by blocking RIG-I signaling. *Eur. J. Pharmacol.* 860:172543. 10.1016/j.ejphar.2019.172543 31323223

[B49] Zhu Y. Zuo W. Chen L. Bian S. Jing J. Gan C. (2019). Repurposing of the anti-helminthic drug niclosamide to treat melanoma and pulmonary metastasis *via* the STAT3 signaling pathway. *Biochem. Pharmacol.* 169:113610. 10.1016/j.bcp.2019.08.012 31465777

[B50] Ziogas D. C. Konstantinou F. Bouros S. Theochari M. Gogas H. (2021). Combining BRAF/MEK inhibitors with immunotherapy in the treatment of metastatic melanoma. *Am. J. Clin. Dermatol.* 22 301–314. 10.1007/s40257-021-00593-9 33765322

