# Supplementary material for: Identification of an ergosterol derivative with anti-melanoma effect from the sponge-derived fungus Pestalotiopsis sp. XWS03F09
Source: Front Microbiol. 2022 Oct 12;13:1008053. doi: 10.3389/fmicb.2022.1008053 (PMC9608767; doi:10.3389/fmicb.2022.1008053)
Supplement: Supplementary file 1 [file Data_Sheet_1.docx]

Supplementary Material

Identification of an ergosterol derivative with anti-melanoma effect from the sponge-derived fungus *Pestalotiopsis* sp. XWS03F09

Tong Xia^1†^, Hui Lei^2†^, Jianv Wang^1^, Yijing He^3^, Hailan Wang^4^, Lanyang Gao^3^, Tingting Qi^5^, Xia Xiong^1*^, Li Liu^1*^, Yongxia Zhu^5*^

Department of Dermatology, The Affiliated Hospital of Southwest Medical University, Luzhou, China

^2^ School of Pharmacy, Southwest Medical University, Luzhou, China

^3^ Department of Science and Technology, The afﬁliated Hospital of Southwest Medical University, Luzhou, China

^4^ School of Public Health, Southwest Medical University, Luzhou, China

^5^ Department of Clinical Pharmacy, Sichuan Cancer Hospital & Institute, Sichuan Cancer Center, School of Medicine, University of Electronic Science and Technology of China, Chengdu, China.

*** Correspondence:**yongxia_zhu0409@uestc.edu.cn (Yongxia Zhu); liuli@swmu.edu.cn (Li Liu);

xiongxia789@126.com (X.X).

Table S1. Primers used for qRT-PCR are shown below

Table S2. Identification of the strain

Table S3 ^1^H-NMR (400 MHz) and ^13^C-NMR (100 MHz) data for **LH-1** in CDCl_3_

Table S4 Ethical approval for C57BL/6 mice

Fig. S1. HRESI-MS spectrum of the compound **LH-1**

Fig. S2 ^1^H NMR (400 MHz, CDCl_3_) spectrum of the compound **LH-1**

Fig. S3 ^13^C NMR (100 MHz, CDCl_3_) spectrum of the compound **LH-1**

Fig. S4 DEPT spectrum of the compound **LH-1**

Fig. S5 HSQC spectrum of the compound **LH-1**

Fig. S6 HMBC spectrum of the compound **LH-1**

Fig. S7. COSY spectrum of the compound **LH-1**

Fig. S8 ^1^H NMR (600 MHz, DMSO-*d*_6_) spectrum of the compound **LH-1**

Fig. S9. NOESY spectrum of the compound **LH-1**

Fig. S10 Effect of **LH-1** on tumor cells

Fig. S11 **LH-1** induced cell apoptosis via mitochondria-mediated apoptosis in A375 cells

Fig. S12 Key NOESY correlation of compound LH-1

Table S1. Primers used for qRT-PCR are shown below:

| **Gene** | **Primers** |
| --- | --- |
| *Egr1* | forward,5′-AGTGATGAACGCAAGAGGCA-3′ |
|  | reverse, 5′-TAGCCACTGGGGATGGGTAA-3′ |
| *MYH10* | forward, 5′-AGCTTCGTTCGGGTTGCTAA-3′ |
|  | reverse,5′-GACCACAGATCCAGCGTCTC-3′ |
| *GAPDHS* | forward, 5′-TAGTGTTGTCCAGCTACGGC-3′ |
|  | reverse, 5′-CTCAACCTTGGGTGGAGGTG-3′ |
| *OBSCN* | forward, 5′-GGAGACCATCACCACCGTAG-3′ |
|  | reverse, 5′-TCCCTGGTGGATACAGCCTC-3′ |
| *ZBTB16* | forward, 5′-GTTGGGGGTCAGCTAGAAAGC-3′ |
|  | reverse, 5′-CAGATCCATGATGCTCCCCTG-3′ |
| *Trim71* | forward, 5′-GACGGAAACCTGTTTGGTGC-3′ |
|  | reverse, 5′-TGGGGCCTGTAGCTCACTAT-3′ |
| *GAPDH* | forward, 5′- ACAACTTTGGTATCGTGGAAGG-3′ |
|  | reverse, 5′- GCCATCACGCCACAGTTTC -3′ |

**Table S2. Identification of the strain**

**S2.1 IS730**

**IS730 588bp**

GGTGAACCTGCGGAGGGATCATTATAGAGTTTTCTAAACTCCCAACCCATGTGAACTTACCATTGTTGCCTCGGCAGAAGCTGCTCGGTATACCCTACCTTGGAACGGCCTACCCTGTAGCGCCTTACCCTGGAACGGCTTACCCTGCAACGGCTGCCGGTGGACTACCAAACTCTTGTTATTTTATTGTAATCTGAGCGTCTTATTTTAATAAGTCAAAACTTTCAACAACGGATCTCTTGGTTCTGGCATCGATGAAGAACGCAGCGAAATGCGATAAGTAATGTGAATTGCAGAATTCAGTGAATCATCGAATCTTTGAACGCACATTGCGCCCATTAGTATTCTAGTGGGCATGCCTGTTCGAGCGTCATTTCAACCCTTAAGCCTAGCTTAGTGTTGGGAGCCTACTGCTTTTGCTAGTTGTAGCTCCTGAAATACAACGGCGGATCTGCGATATCCTCTGAGCGTAGTAATTTTTATCTCGCTTTTGACTGGAGTTGCAGCGTCTTTAGCCGCTAAACCCCCCAATTTTTAATGGTTGACCTCGGATCAGGTAGGAATACCCGCTGAACTTAAGCATATCAA

| **Description** | **[Max score](http://blast.ncbi.nlm.nih.gov/Blast.cgi?CMD=Get&ALIGNMENTS=100&ALIGNMENT_VIEW=Pairwise&DATABASE_SORT=0&DESCRIPTIONS=100&DYNAMIC_FORMAT=on&FIRST_QUERY_NUM=0&FORMAT_OBJECT=Alignment&FORMAT_PAGE_TARGET=&FORMAT_TYPE=HTML&GET_SEQUENCE=yes&I_THRESH=&LINE_LENGTH=60&MASK_CHAR=2&MASK_COLOR=1&NUM_OVERVIEW=100&OLD_BLAST=false&PAGE=MegaBlast&QUERY_INDEX=0&QUERY_NUMBER=0&RESULTS_PAGE_TARGET=&RID=MJUURCY3015&SHOW_LINKOUT=yes&SHOW_OVERVIEW=yes&STEP_NUMBER=&OLD_VIEW=false&DISPLAY_SORT=1&HSP_SORT=1)** | **[Total score](http://blast.ncbi.nlm.nih.gov/Blast.cgi?CMD=Get&ALIGNMENTS=100&ALIGNMENT_VIEW=Pairwise&DATABASE_SORT=0&DESCRIPTIONS=100&DYNAMIC_FORMAT=on&FIRST_QUERY_NUM=0&FORMAT_OBJECT=Alignment&FORMAT_PAGE_TARGET=&FORMAT_TYPE=HTML&GET_SEQUENCE=yes&I_THRESH=&LINE_LENGTH=60&MASK_CHAR=2&MASK_COLOR=1&NUM_OVERVIEW=100&OLD_BLAST=false&PAGE=MegaBlast&QUERY_INDEX=0&QUERY_NUMBER=0&RESULTS_PAGE_TARGET=&RID=MJUURCY3015&SHOW_LINKOUT=yes&SHOW_OVERVIEW=yes&STEP_NUMBER=&OLD_VIEW=false&DISPLAY_SORT=2&HSP_SORT=1)** | **[Query cover](http://blast.ncbi.nlm.nih.gov/Blast.cgi?CMD=Get&ALIGNMENTS=100&ALIGNMENT_VIEW=Pairwise&DATABASE_SORT=0&DESCRIPTIONS=100&DYNAMIC_FORMAT=on&FIRST_QUERY_NUM=0&FORMAT_OBJECT=Alignment&FORMAT_PAGE_TARGET=&FORMAT_TYPE=HTML&GET_SEQUENCE=yes&I_THRESH=&LINE_LENGTH=60&MASK_CHAR=2&MASK_COLOR=1&NUM_OVERVIEW=100&OLD_BLAST=false&PAGE=MegaBlast&QUERY_INDEX=0&QUERY_NUMBER=0&RESULTS_PAGE_TARGET=&RID=MJUURCY3015&SHOW_LINKOUT=yes&SHOW_OVERVIEW=yes&STEP_NUMBER=&OLD_VIEW=false&DISPLAY_SORT=4&HSP_SORT=0)** | **[E value](http://blast.ncbi.nlm.nih.gov/Blast.cgi?CMD=Get&ALIGNMENTS=100&ALIGNMENT_VIEW=Pairwise&DATABASE_SORT=0&DESCRIPTIONS=100&DYNAMIC_FORMAT=on&FIRST_QUERY_NUM=0&FORMAT_OBJECT=Alignment&FORMAT_PAGE_TARGET=&FORMAT_TYPE=HTML&GET_SEQUENCE=yes&I_THRESH=&LINE_LENGTH=60&MASK_CHAR=2&MASK_COLOR=1&NUM_OVERVIEW=100&OLD_BLAST=false&PAGE=MegaBlast&QUERY_INDEX=0&QUERY_NUMBER=0&RESULTS_PAGE_TARGET=&RID=MJUURCY3015&SHOW_LINKOUT=yes&SHOW_OVERVIEW=yes&STEP_NUMBER=&OLD_VIEW=false&DISPLAY_SORT=0&HSP_SORT=0)** | **[Ident](http://blast.ncbi.nlm.nih.gov/Blast.cgi?CMD=Get&ALIGNMENTS=100&ALIGNMENT_VIEW=Pairwise&DATABASE_SORT=0&DESCRIPTIONS=100&DYNAMIC_FORMAT=on&FIRST_QUERY_NUM=0&FORMAT_OBJECT=Alignment&FORMAT_PAGE_TARGET=&FORMAT_TYPE=HTML&GET_SEQUENCE=yes&I_THRESH=&LINE_LENGTH=60&MASK_CHAR=2&MASK_COLOR=1&NUM_OVERVIEW=100&OLD_BLAST=false&PAGE=MegaBlast&QUERY_INDEX=0&QUERY_NUMBER=0&RESULTS_PAGE_TARGET=&RID=MJUURCY3015&SHOW_LINKOUT=yes&SHOW_OVERVIEW=yes&STEP_NUMBER=&DISPLAY_SORT=3&HSP_SORT=3)** | **Accession** |
| --- | --- | --- | --- | --- | --- | --- |
| [Pestalotiopsis neglecta isolate H4234 18S ribosomal RNA gene, partial sequence; internal transcribed spacer 1, 5.8S ribosomal RNA gene, and internal transcribed spacer 2, complete sequence; and 28S ribosomal RNA gene, partial sequence](http://blast.ncbi.nlm.nih.gov/Blast.cgi#alnHdr_291293636) | 1081 | 1081 | 100% | 0.0 | 99.0% | [GU595050.1](http://www.ncbi.nlm.nih.gov/nucleotide/291293636?report=genbank&log$=nucltop&blast_rank=1&RID=MJUURCY3015) |
| [Pestalotiopsis heterocornis strain 3.9157 internal transcribed spacer 1, partial sequence; 5.8S ribosomal RNA gene, complete sequence; and internal transcribed spacer 2, partial sequence](http://blast.ncbi.nlm.nih.gov/Blast.cgi#alnHdr_359294534) | 1075 | 1075 | 98% | 0.0 | 99.9% | KM199307.1 |
| [Pestalotiopsis sp. 7-6 18S ribosomal RNA gene, partial sequence; internal transcribed spacer 1, 5.8S ribosomal RNA gene, and internal transcribed spacer 2, complete sequence; and 28S ribosomal RNA gene, partial sequence](http://blast.ncbi.nlm.nih.gov/Blast.cgi#alnHdr_312205587) | 1074 | 1074 | 100% | 0.0 | 99.0% | [HM486429.1](http://www.ncbi.nlm.nih.gov/nucleotide/312205587?report=genbank&log$=nucltop&blast_rank=3&RID=MJUURCY3015) |
| [Pestalotiopsis sp. EN 12 (HKUCC 8321) 18S ribosomal RNA gene, partial sequence; internal transcribed spacer 1, 5.8S ribosomal RNA gene and internal transcribed spacer 2, complete sequence; and 28S ribosomal RNA gene, partial sequence](http://blast.ncbi.nlm.nih.gov/Blast.cgi" \l "alnHdr_24417621" \o "Go to alignment for Pestalotiopsis sp. EN 12 (HKUCC 8321) 18S ribosomal RNA gene, partial sequence; internal transcribed spacer 1, 5.8S ribosomal RNA gene and internal transcribed spacer 2, complete sequence; and 28S ribosomal RNA gene, partial sequence) | 1061 | 1061 | 98% | 0.0 | 99.0% | [AF409994.1](http://www.ncbi.nlm.nih.gov/nucleotide/24417621?report=genbank&log$=nucltop&blast_rank=4&RID=MJUURCY3015) |
| [Pestalotiopsis colombiensis strain CBS 118553 18S ribosomal RNA gene, partial sequence; internal transcribed spacer 1, 5.8S ribosomal RNA gene, and internal transcribed spacer 2, complete sequence; and 28S ribosomal RNA gene, partial sequence](http://blast.ncbi.nlm.nih.gov/Blast.cgi#alnHdr_698175439) | 1059 | 1059 | 99% | 0.0 | 99.0% |  |
| [Pestalotiopsis adusta strain LPJZ02 18S ribosomal RNA gene, partial sequence; internal transcribed spacer 1, 5.8S ribosomal RNA gene, and internal transcribed spacer 2, complete sequence; and 28S ribosomal RNA gene, partial sequence](http://blast.ncbi.nlm.nih.gov/Blast.cgi#alnHdr_700599961) | 1055 | 1055 | 99% | 0.0 | 99.0% | [KJ885548.2](http://www.ncbi.nlm.nih.gov/nucleotide/700599961?report=genbank&log$=nucltop&blast_rank=6&RID=MJUURCY3015) |
| [Pestalotiopsis diploclisia strain CBS 115449 18S ribosomal RNA gene, partial sequence; internal transcribed spacer 1, 5.8S ribosomal RNA gene, and internal transcribed spacer 2, complete sequence; and 28S ribosomal RNA gene, partial sequence](http://blast.ncbi.nlm.nih.gov/Blast.cgi#alnHdr_698175446) | 1053 | 1053 | 100% | 0.0 | 99.0% | [KM199314.1](http://www.ncbi.nlm.nih.gov/nucleotide/698175446?report=genbank&log$=nucltop&blast_rank=7&RID=MJUURCY3015) |
| [Pestalotiopsis microspora strain PE06 18S ribosomal RNA gene, partial sequence; internal transcribed spacer 1, 5.8S ribosomal RNA gene, and internal transcribed spacer 2, complete sequence; and 28S ribosomal RNA gene, partial sequence](http://blast.ncbi.nlm.nih.gov/Blast.cgi#alnHdr_269978425) | 1053 | 1053 | 100% | 0.0 | 99.0% | [GU171385.1](http://www.ncbi.nlm.nih.gov/nucleotide/269978425?report=genbank&log$=nucltop&blast_rank=8&RID=MJUURCY3015) |
| [Pestalotiopsis neglecta strain LK29 18S ribosomal RNA gene, partial sequence; internal transcribed spacer 1, 5.8S ribosomal RNA gene, and internal transcribed spacer 2, complete sequence; and 28S ribosomal RNA gene, partial sequence](http://blast.ncbi.nlm.nih.gov/Blast.cgi#alnHdr_62996933) | 1053 | 1053 | 100% | 0.0 | 99.0% | [DQ000992.1](http://www.ncbi.nlm.nih.gov/nucleotide/62996933?report=genbank&log$=nucltop&blast_rank=9&RID=MJUURCY3015) |

**S2.2 PCR**


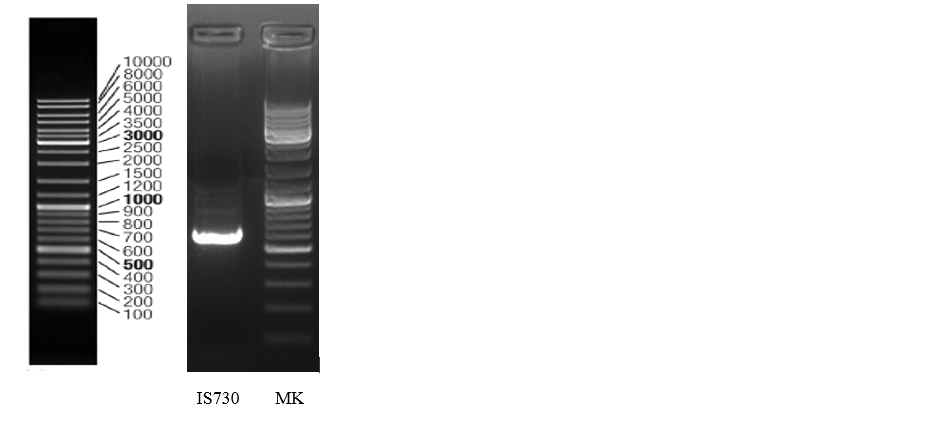


Table S3 ^1^H-NMR (400 MHz) and ^13^C-NMR (100 MHz) data for **LH-1** in CDCl_3_

|  | **LH-1** | | | | | |
| --- | --- | --- | --- | --- | --- | --- |
| **No.** | *δ*_C_ | *δ*_H_ **(*J* in Hz)** |  | **No.** | *δ*_C_ *δ***_H_ (*J* in Hz)** | |
| **1** | 35.8 | 1.48 |  | **16** | 28.8 | 1.6, m |
| **2** | 28.5 | 1.31, m, 1.92, m |  | **17** | 54.7 | 1.15, m |
| **3** | 79.0 | 3.24, dd (11.7, 4.4) | | **18** | 11.3 | 0.61, s |
| **4** | 39.0 | - |  | **19** | 18.5 | 1.00, s |
| **5** | 50.2 | 1.12, m |  | **20** | 36.3 | 1.48, m |
| **6** | 22.0 | 1.02, m |  | **21** | 18.7 | 0.96, d (6.5) |
| **7** | 28.0 | 1.58, m, 1.68, m |  | **22** | 34.7 | 1.32, m |
| **8** | 127.9 | - |  | **23** | 31.1 | 1.98, m |
| **9** | 135.9 | - |  | **24** | 156.9 | - |
| **10** | 37.0 | 1.37, m |  | **25** | 33.8 | 2.21, m |
| **11** | 22.1 | 2.05, m |  | **26** | 19.9 | 0.99, d (6.5) |
| **12** | 37.0 | 1.96, m |  | **27** | 21.9 | 0.95, d (6.5) |
| **13** | 42.7 | - |  | **28** | 105.9 | 4.71, br s, 4.66, br s |
| **14** | 51.9 | 2.02, m |  | **29** | 15.4 | 0.81,s |
| **15** | 23.8 | 1.42, m |  | **30** | 27.9 | 1.02, s |

Table S4 Ethical approval for C57BL/6 mice


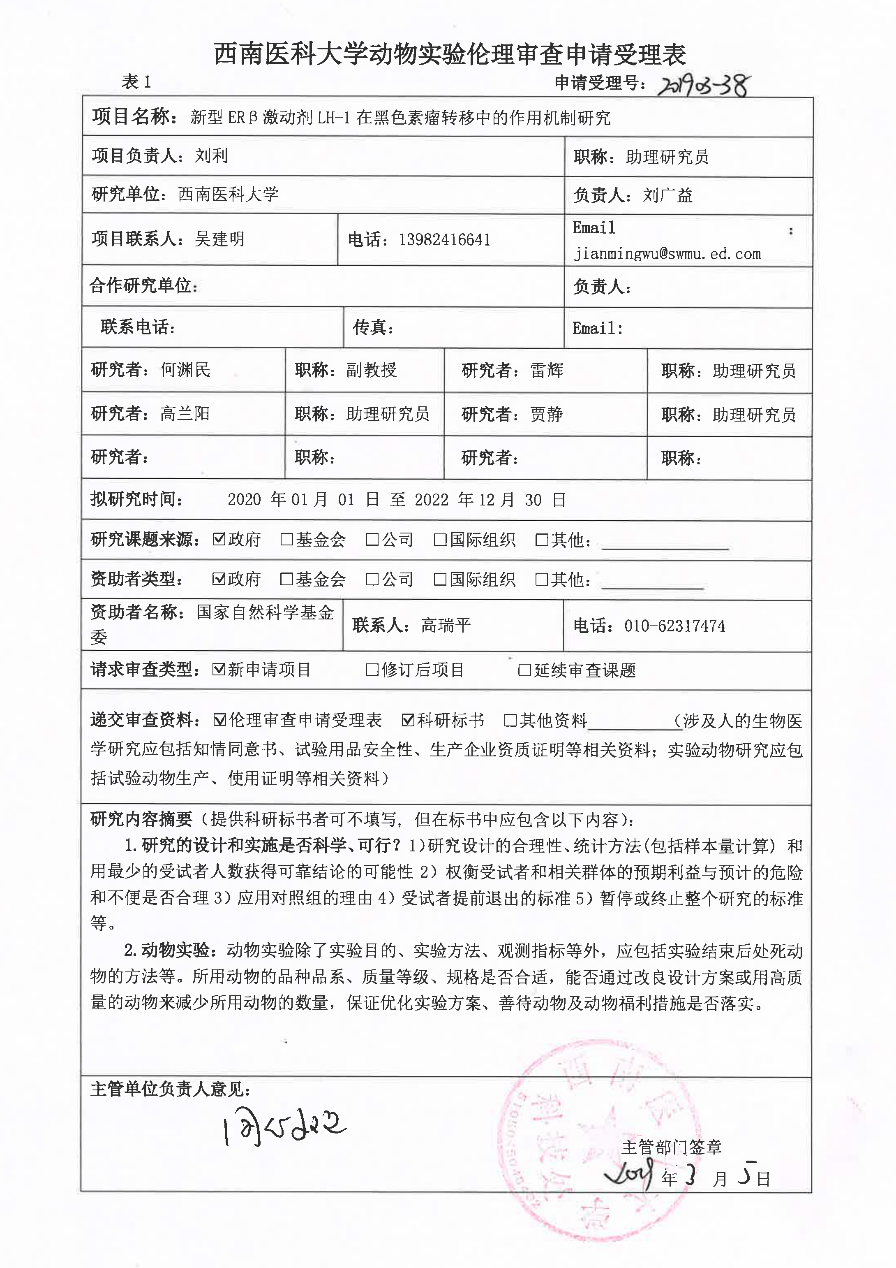

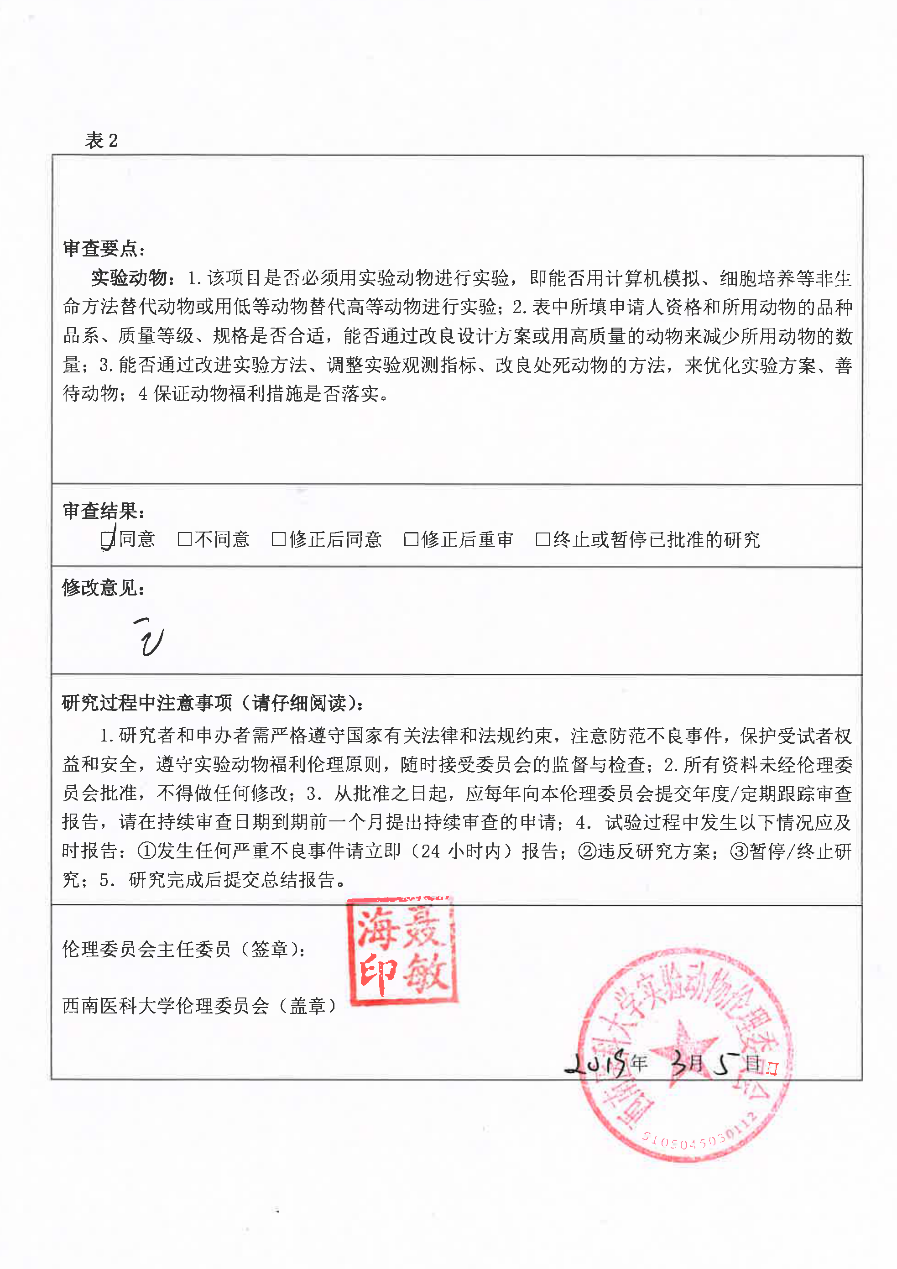


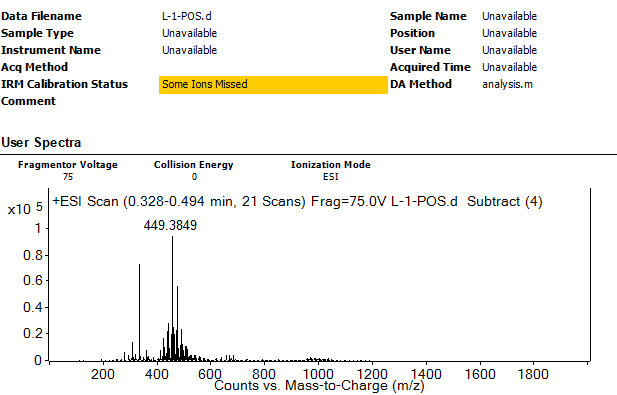


Fig. S1. HRESI-MS spectrum of the compound **LH-1**


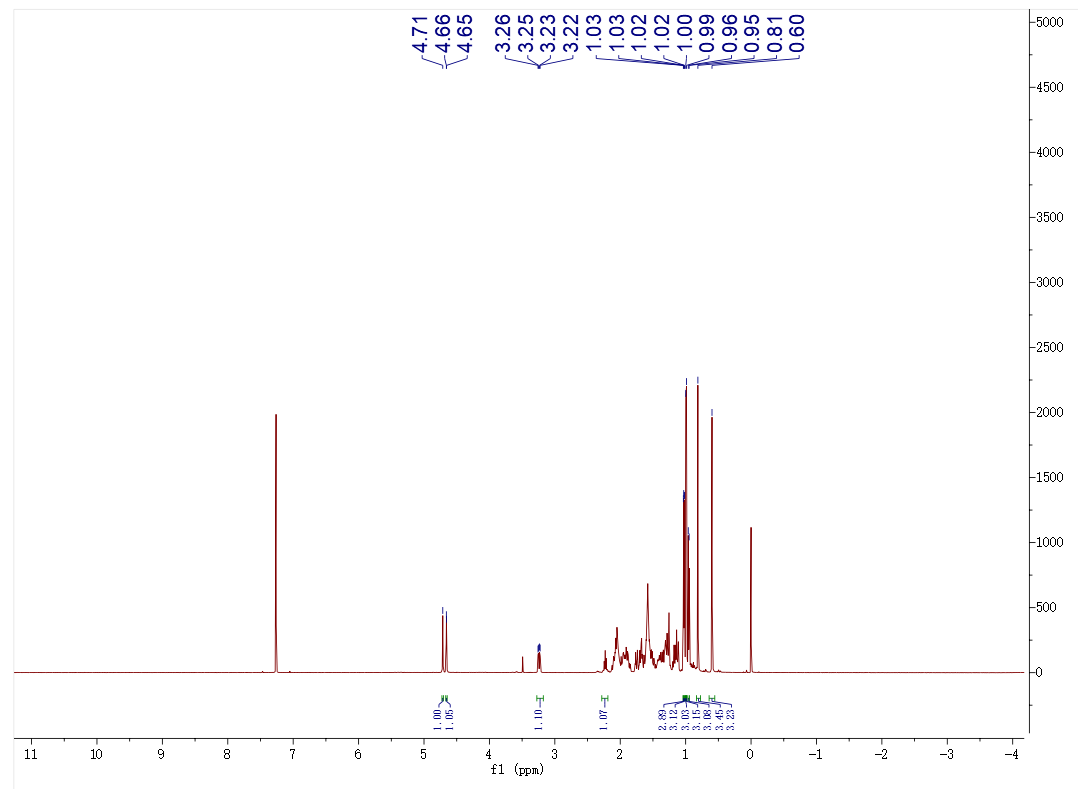


Fig. S2 ^1^H NMR (400 MHz, CDCl_3_) spectrum of the compound **LH-1**


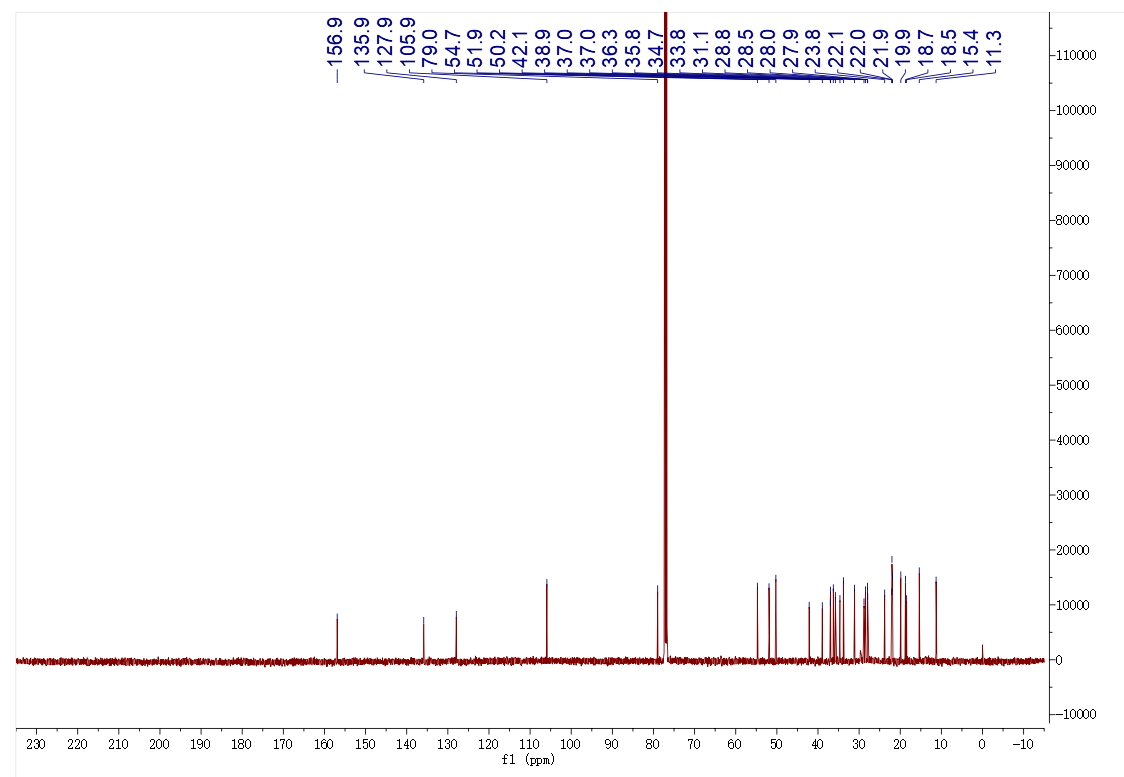


Fig. S3 ^13^C NMR (100 MHz, CDCl_3_) spectrum of the compound **LH-1**


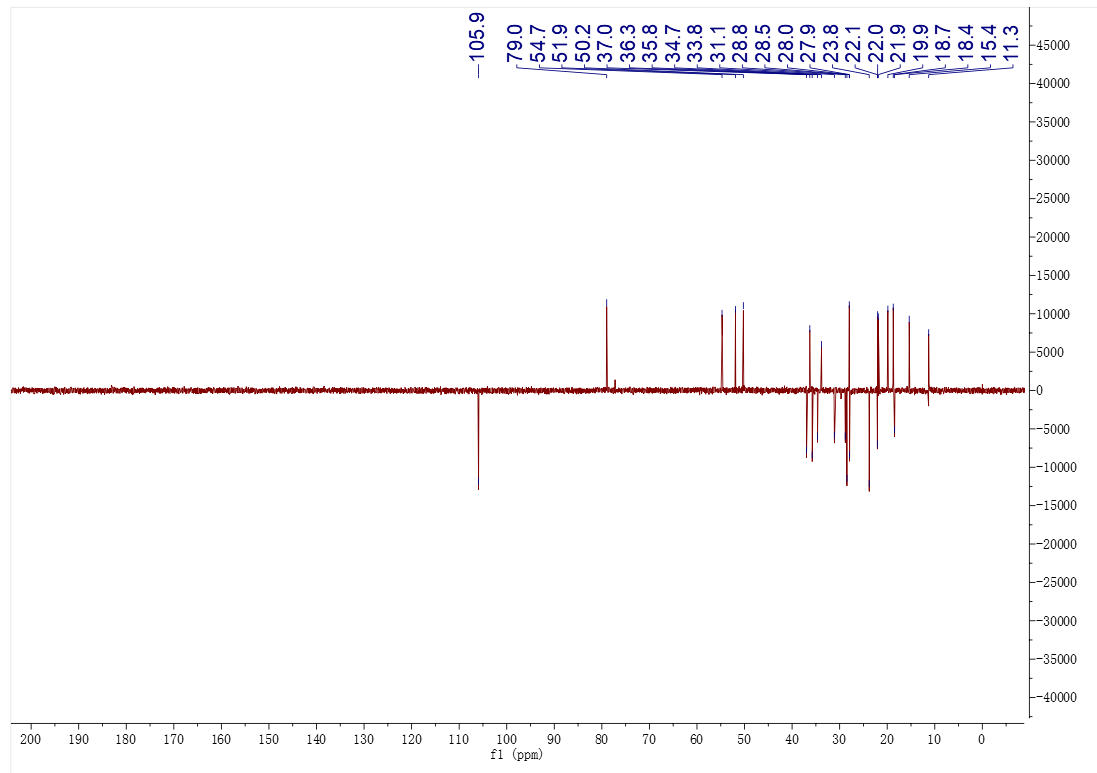


Fig. S4 DEPT spectrum of the compound **LH-1**


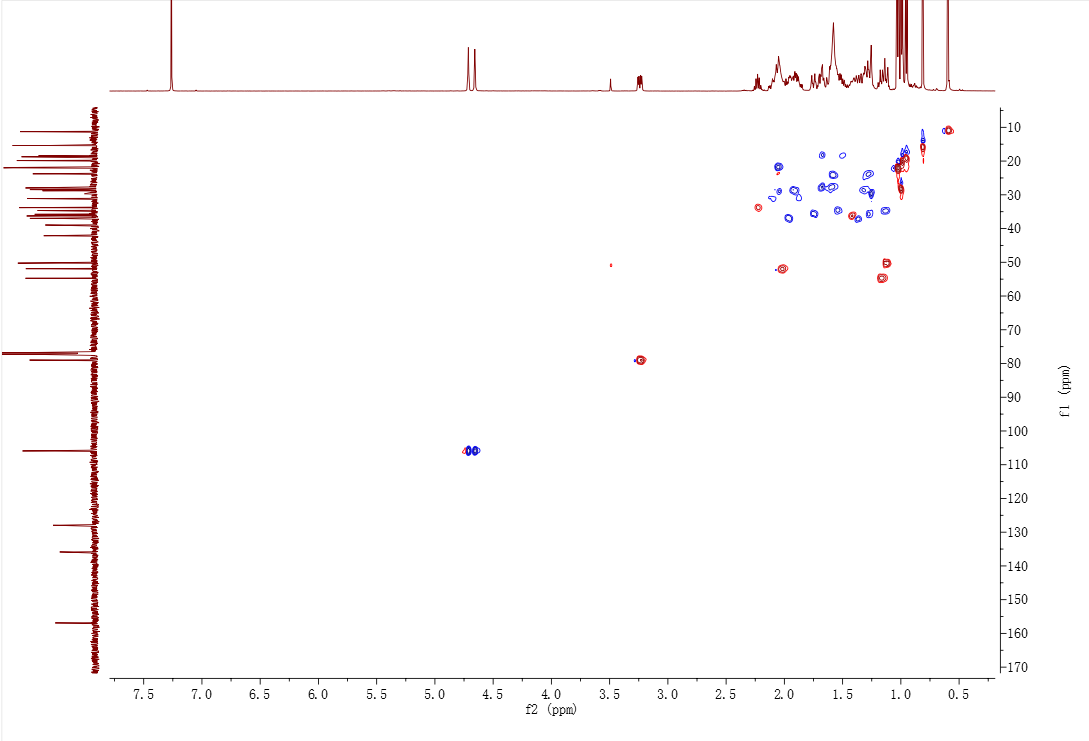


Fig. S5 HSQC spectrum of the compound **LH-1**


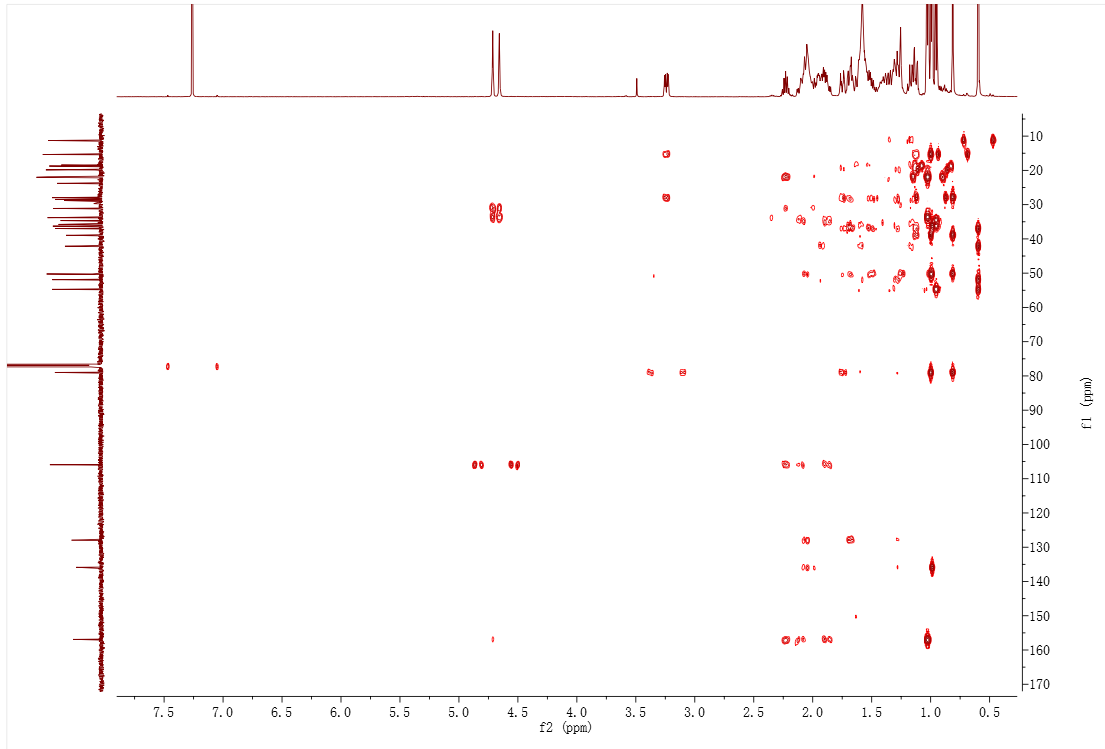


Fig. S6 HMBC spectrum of the compound **LH-1**


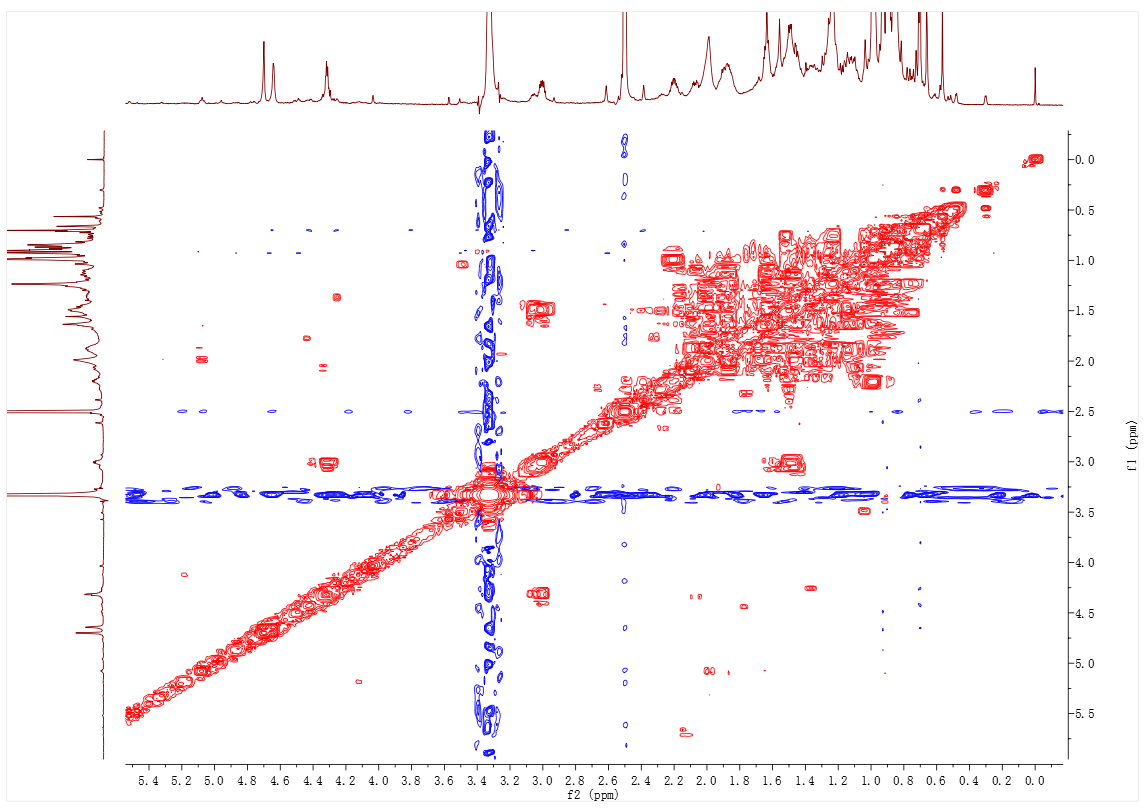


Fig. S7. COSY spectrum of the compound **LH-1**


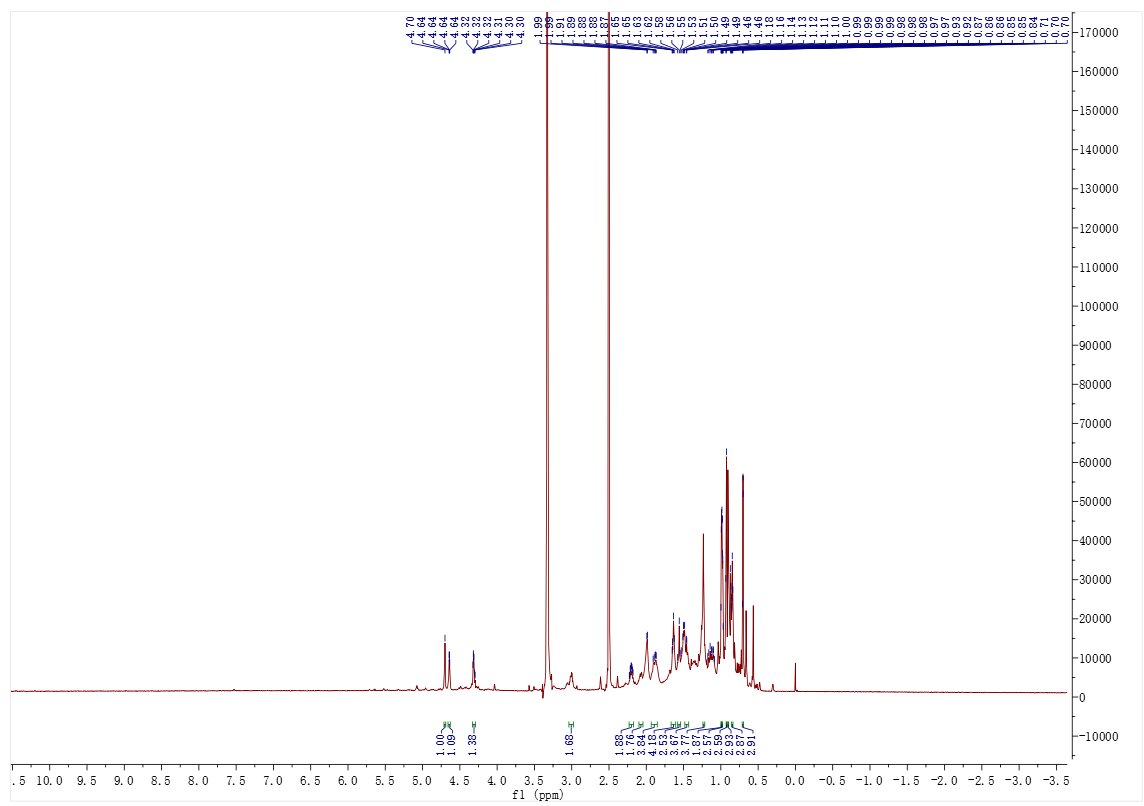


Fig. S8 ^1^H NMR (600 MHz, DMSO-*d*_6_) spectrum of the compound **LH-1**


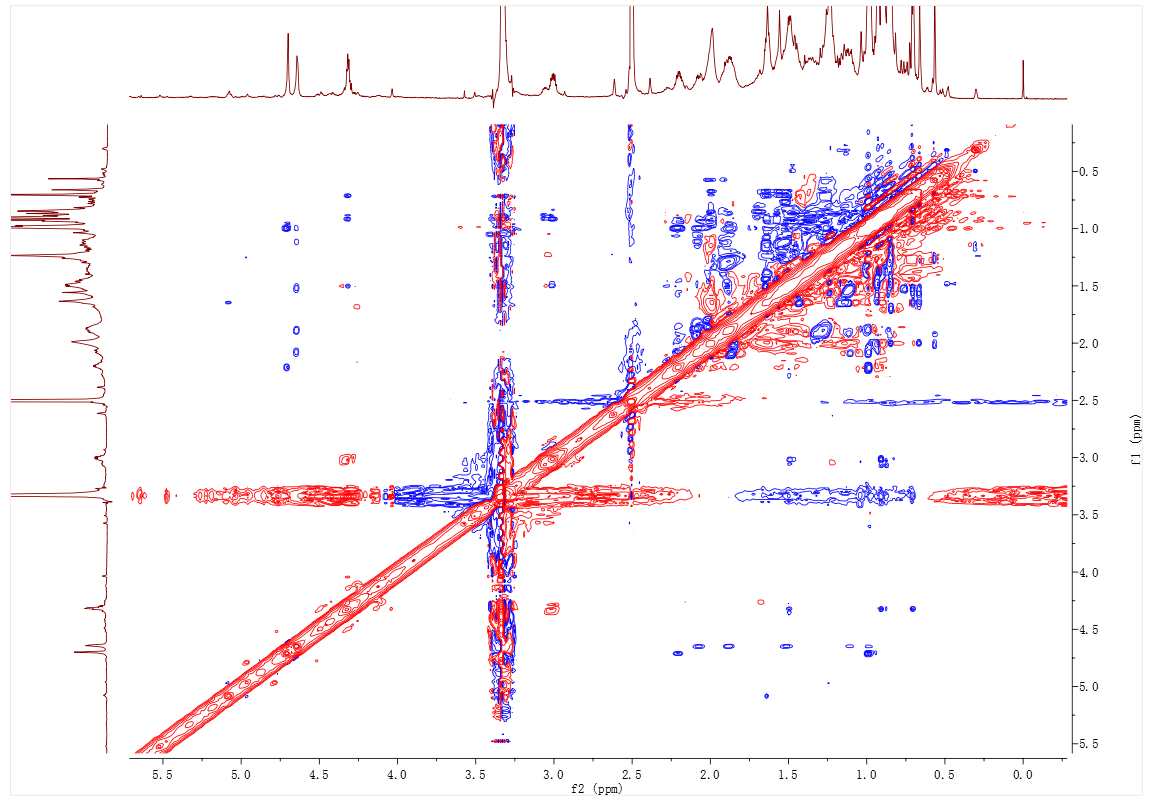


Fig. S9. NOESY spectrum of the compound **LH-1**


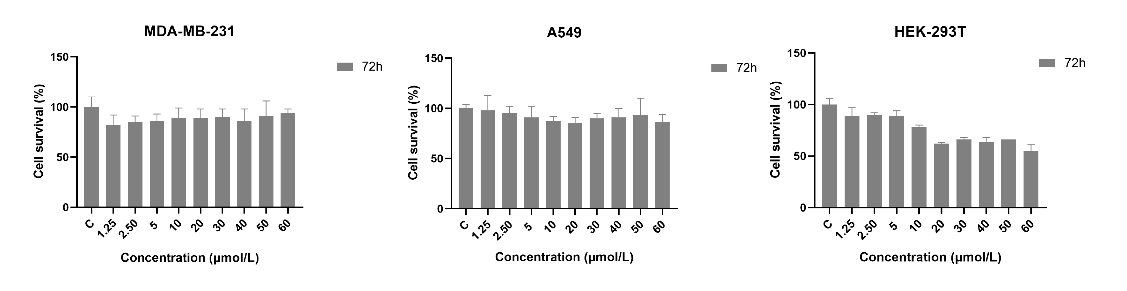


Fig. S10 Effect of **LH-1** on tumor cells. Human breast cancer cell MDA-MB-231, human carcinoma cell A549 and human renal epithelial cell HEK-293T were treated with different concentrations of LH-1 for 72 h respectively. And the viability was measured by the MTT assay.


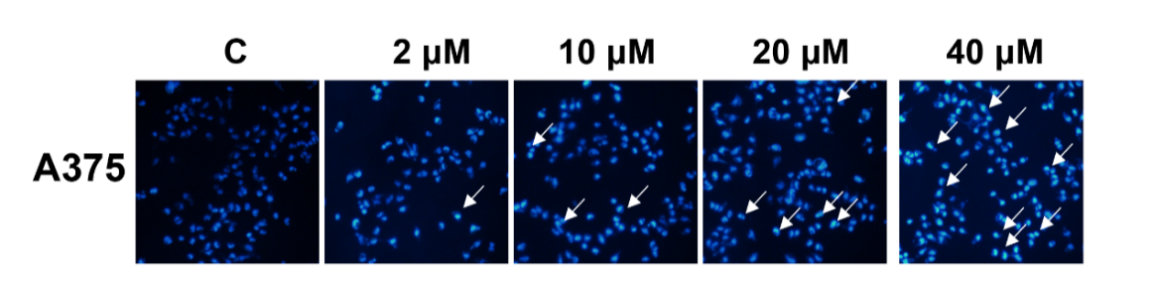


Fig. S11 **LH-1** induced cell apoptosis via mitochondria-mediated apoptosis. In A375 cells, Hoechst staining was used to stain apoptotic cells after treatment with **LH-1**.


 NOESY

Fig. S12 Key NOESY correlation of compound **LH-1**
